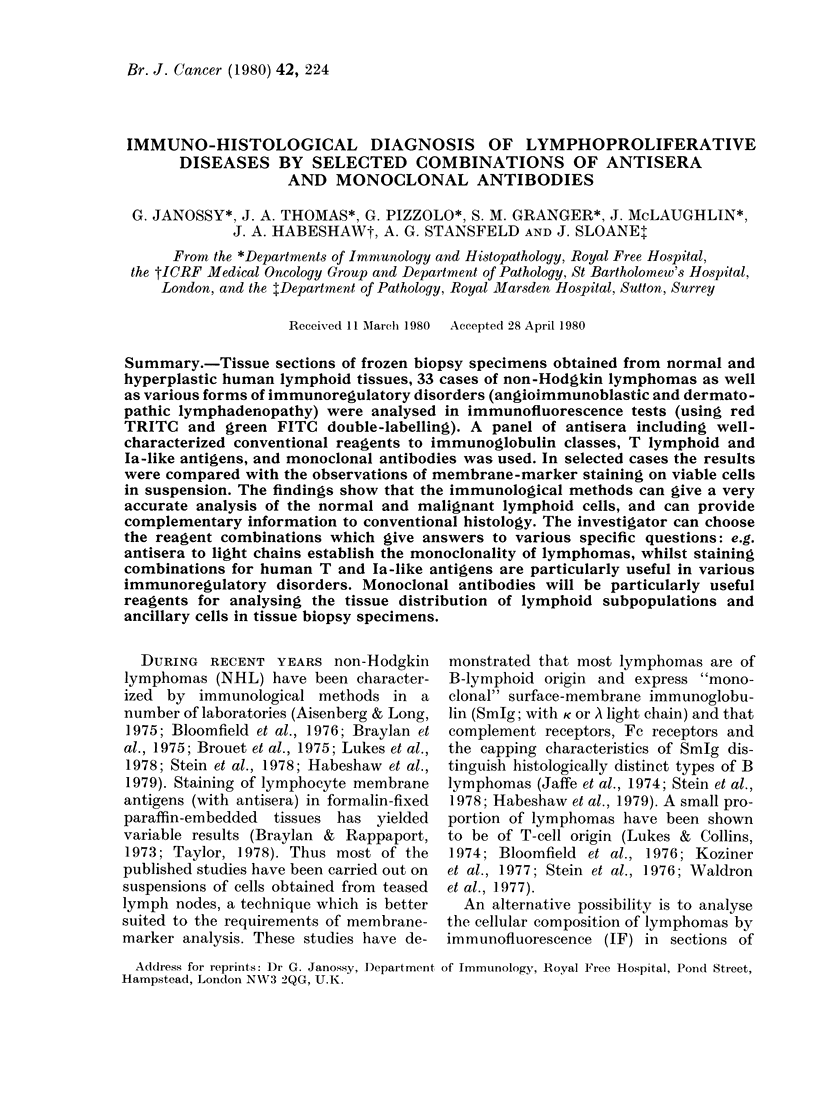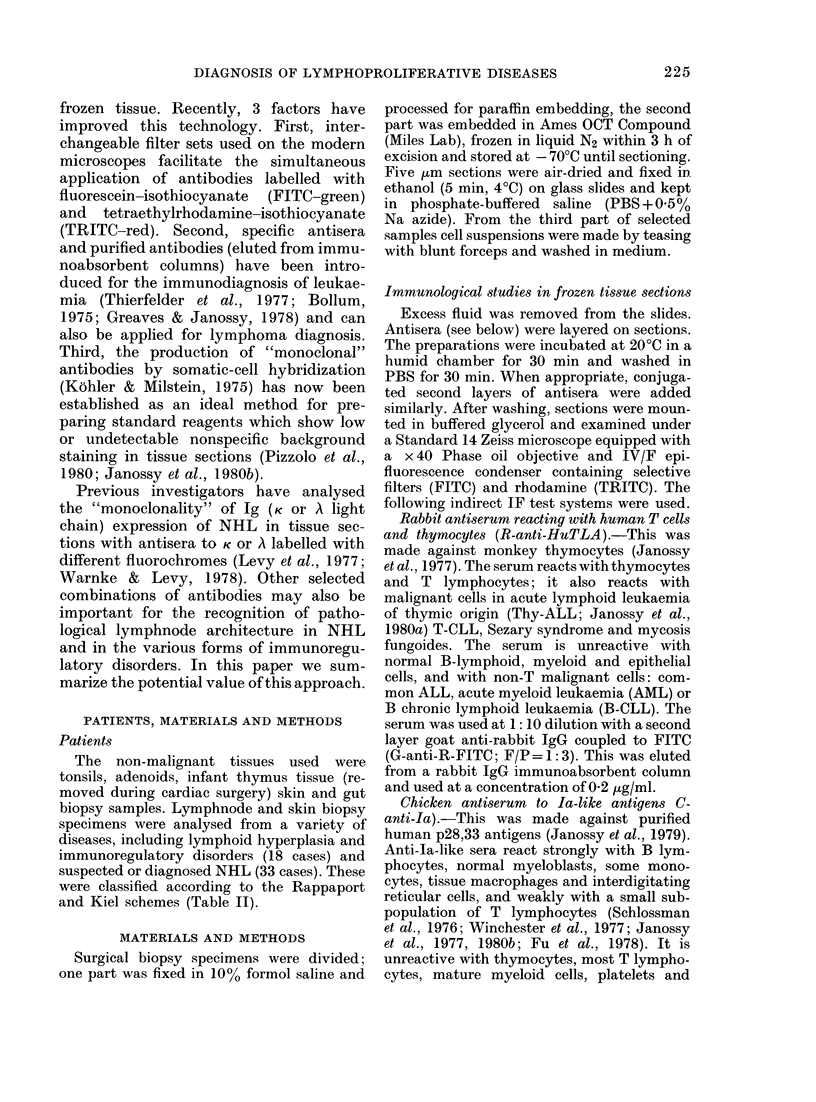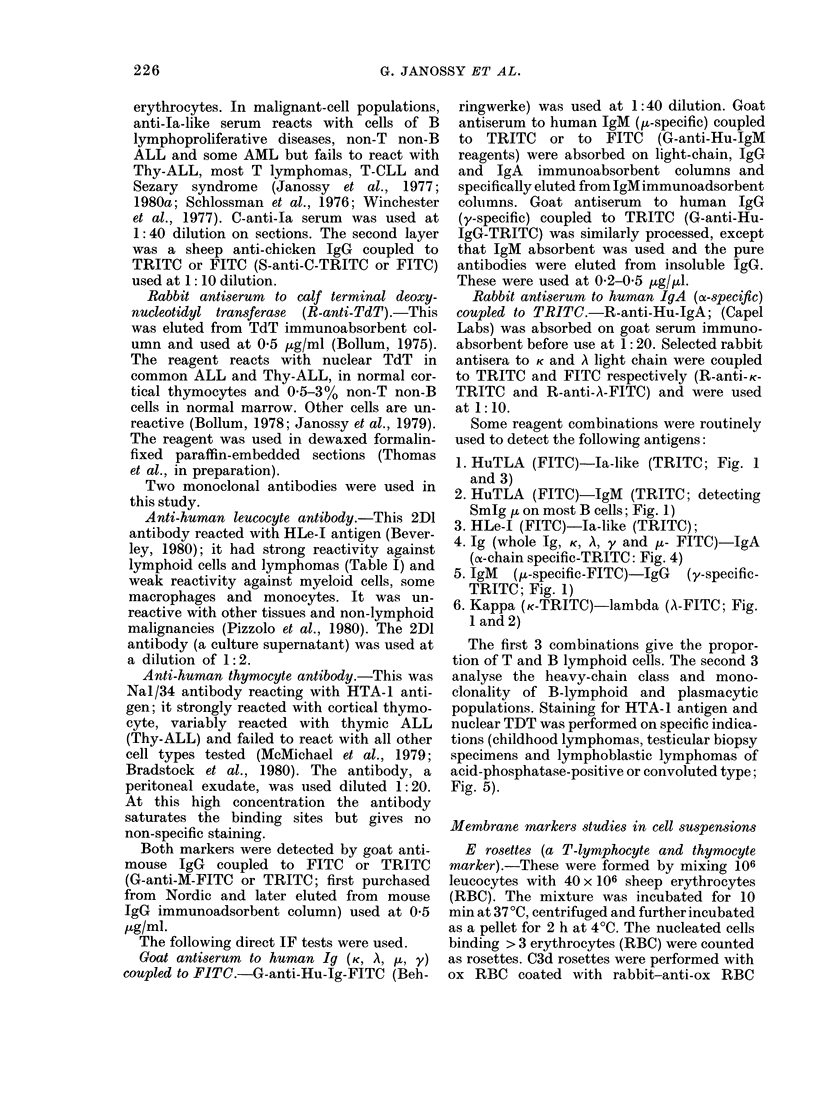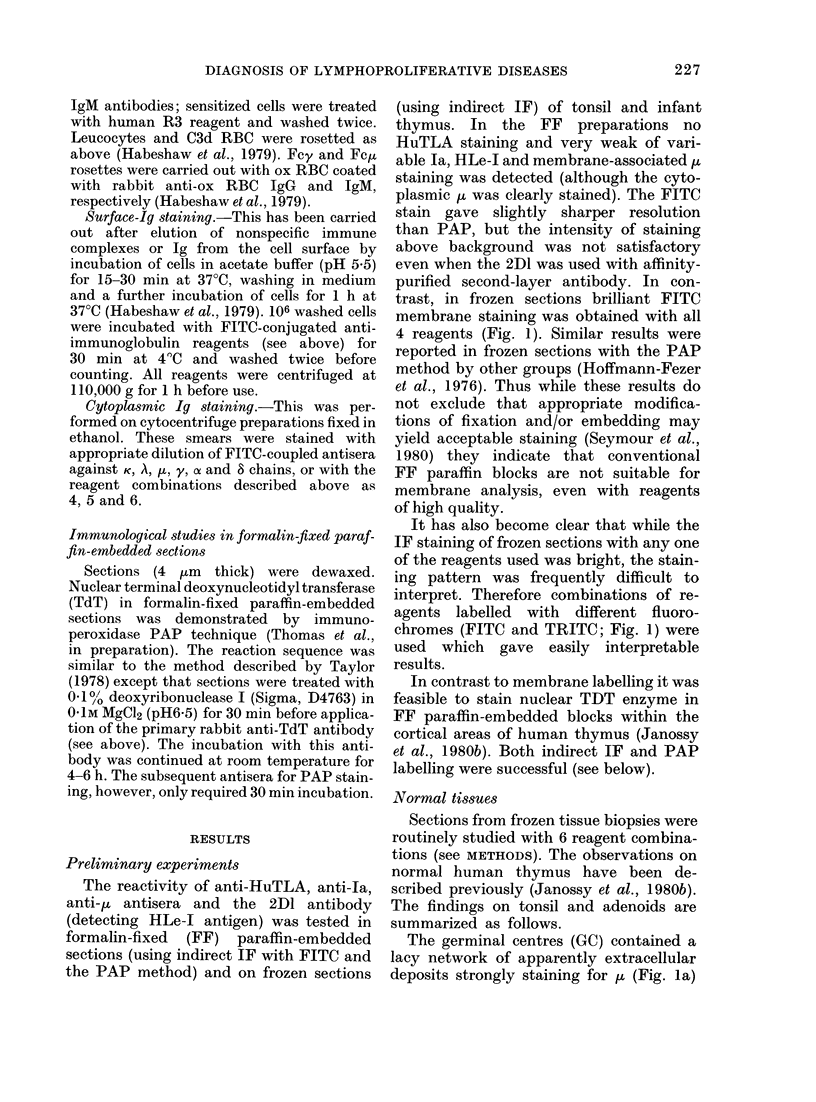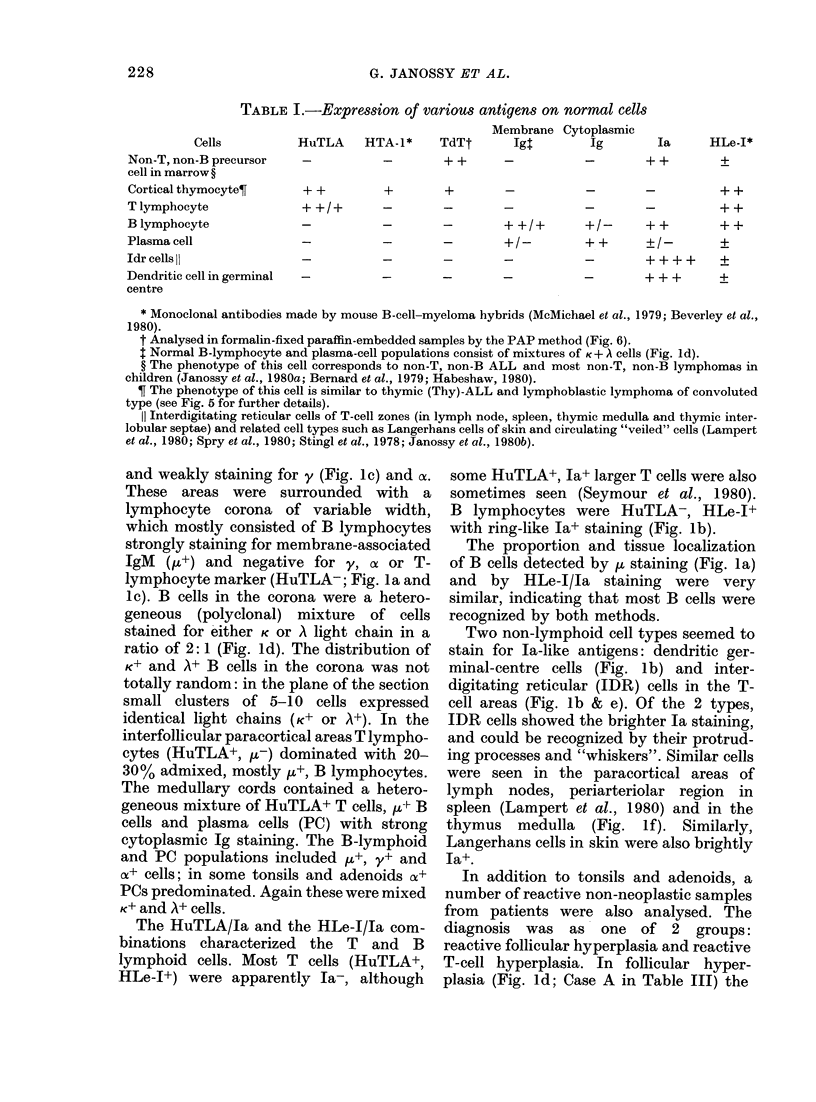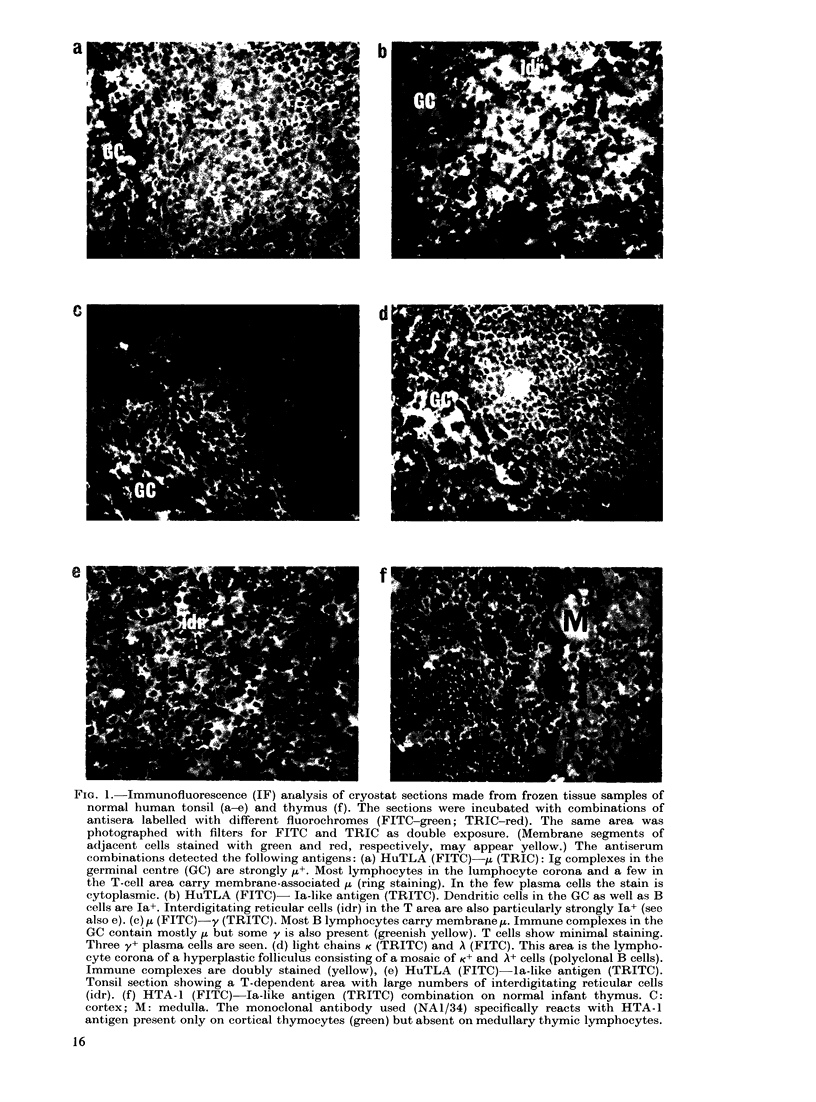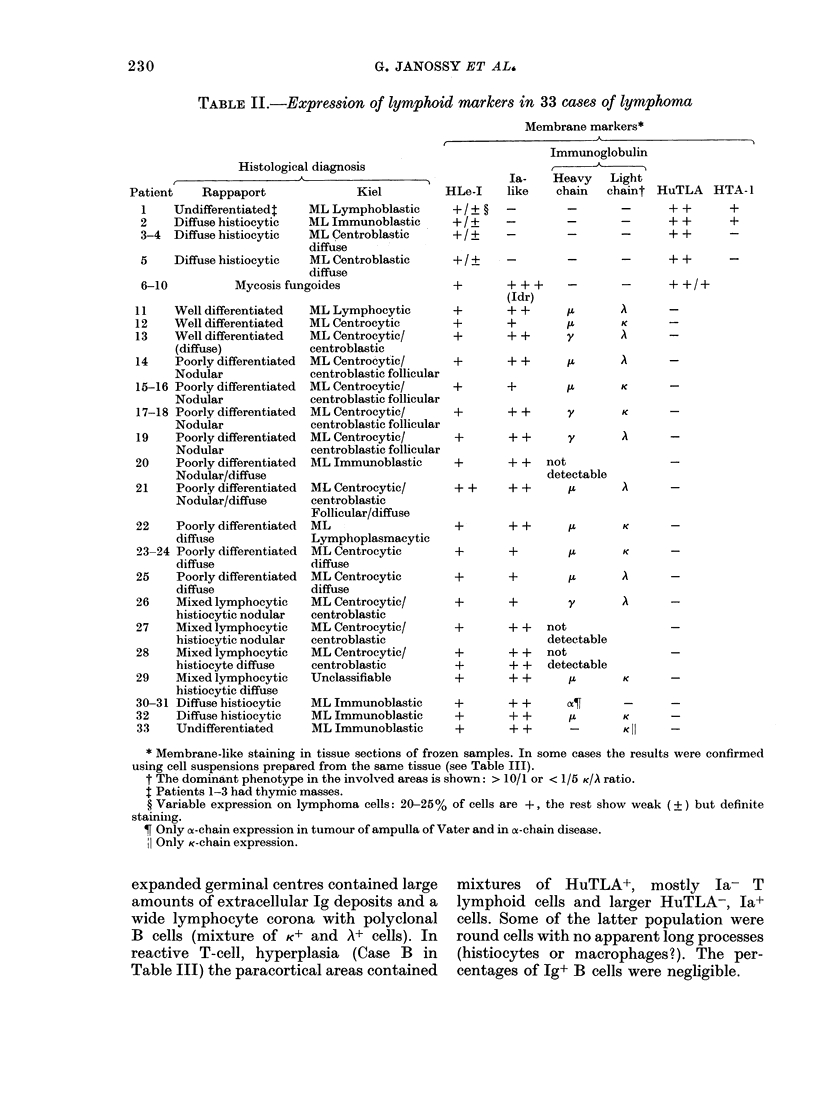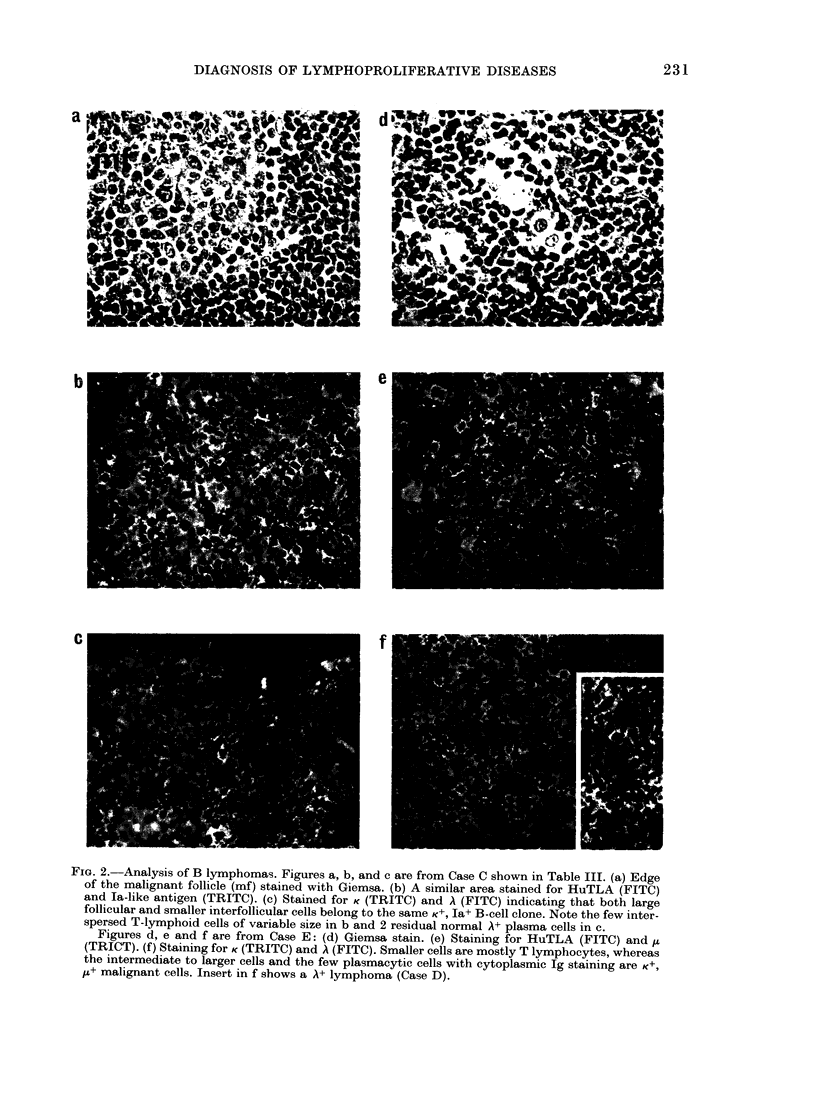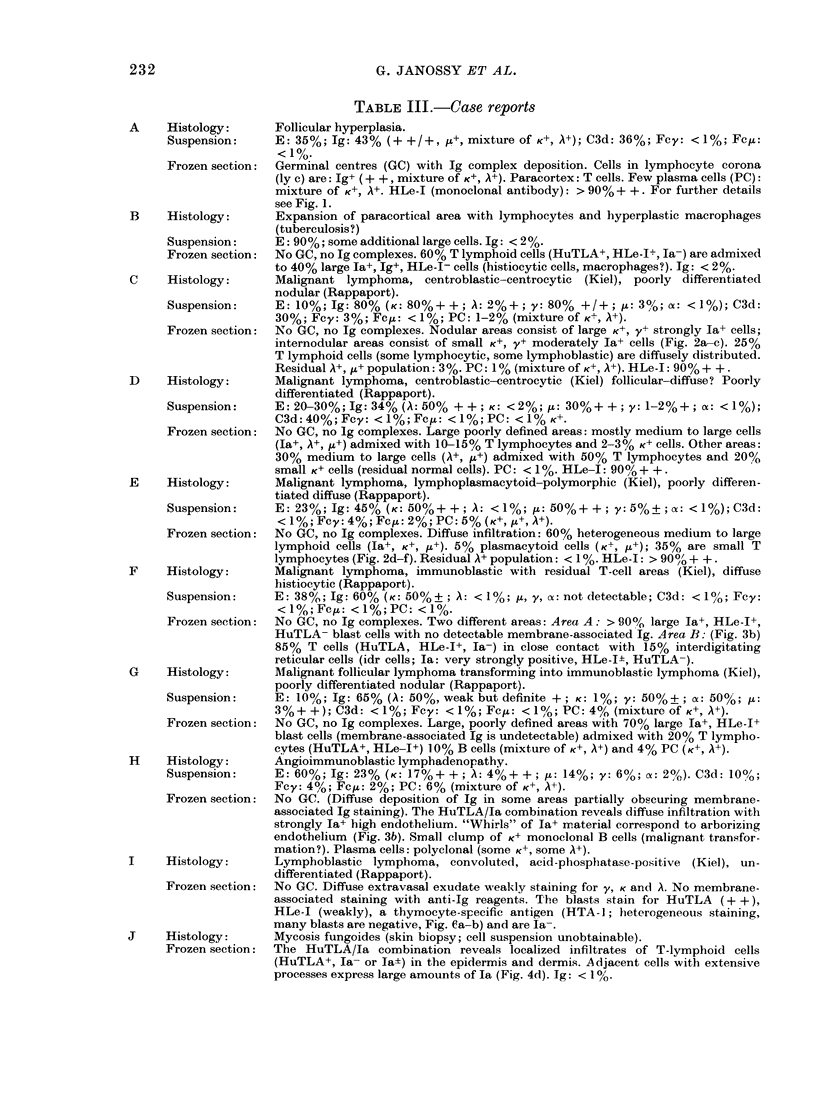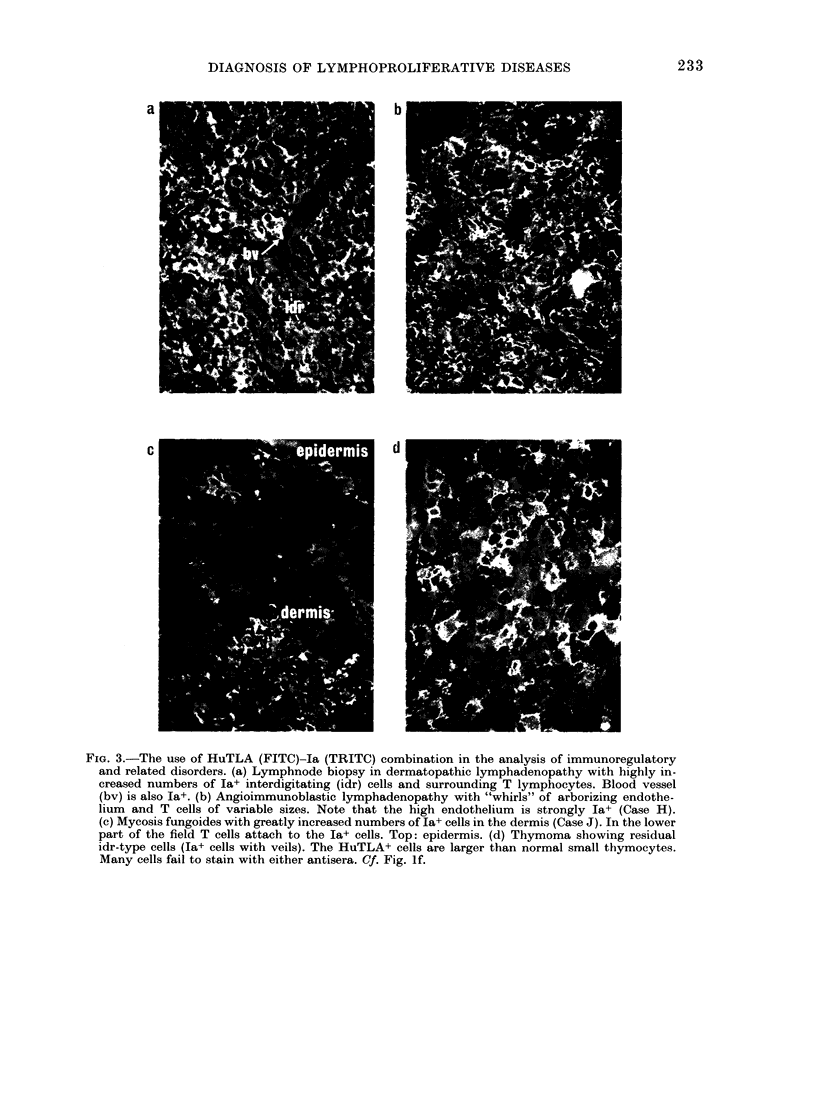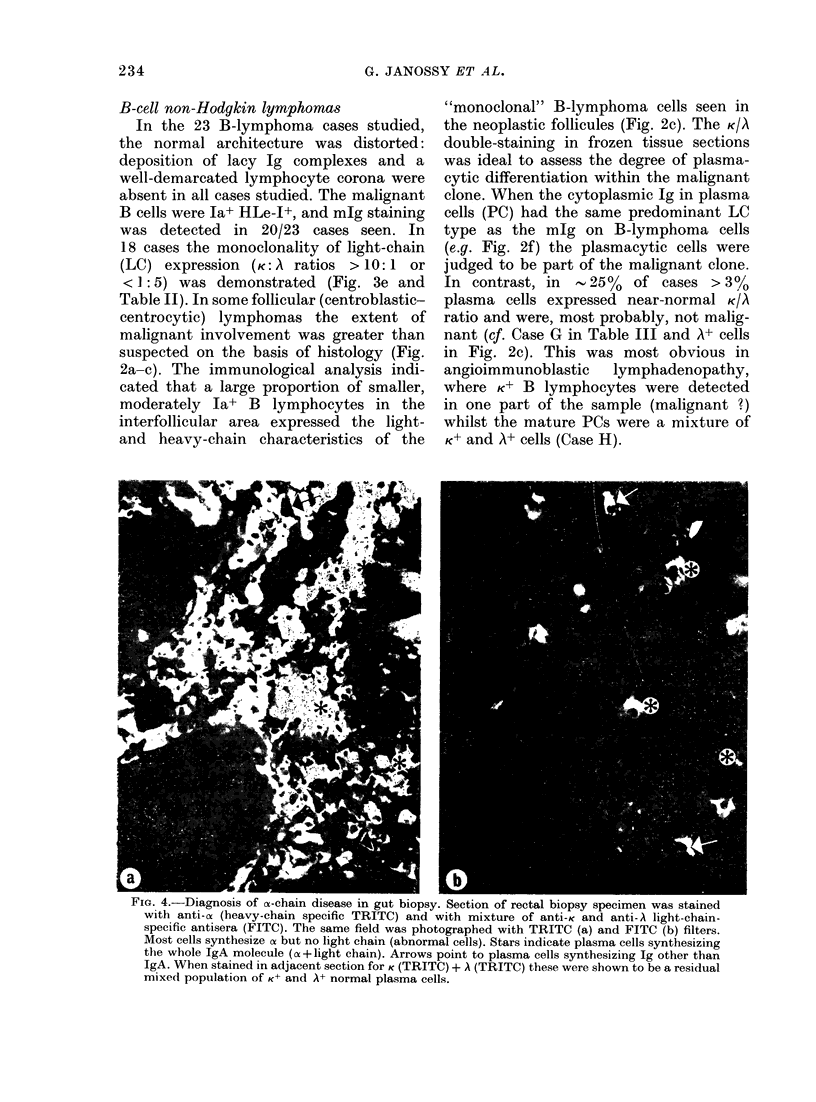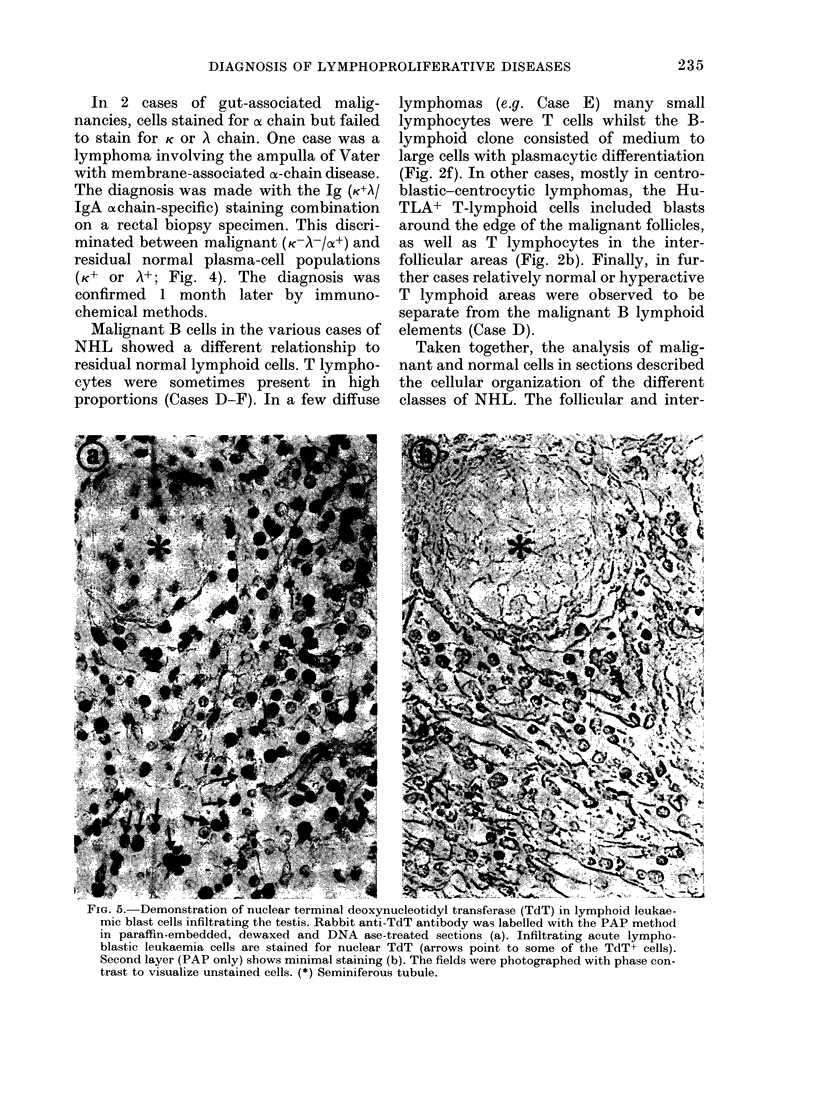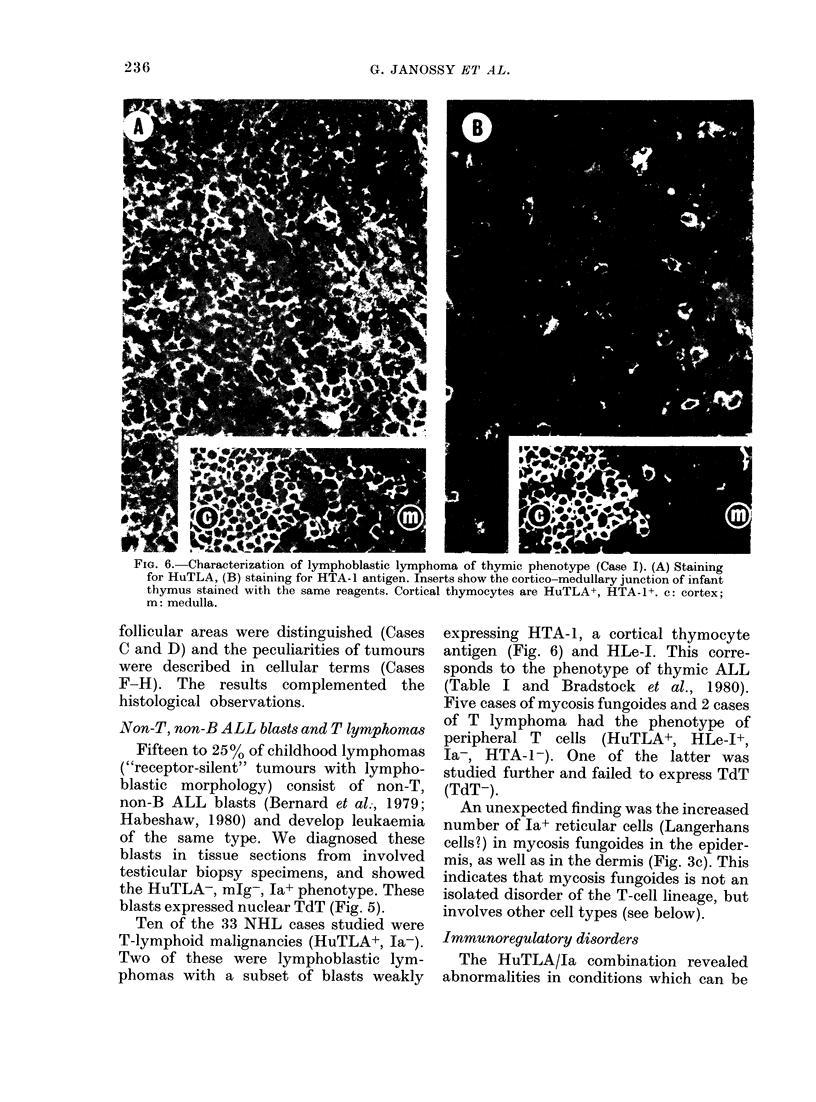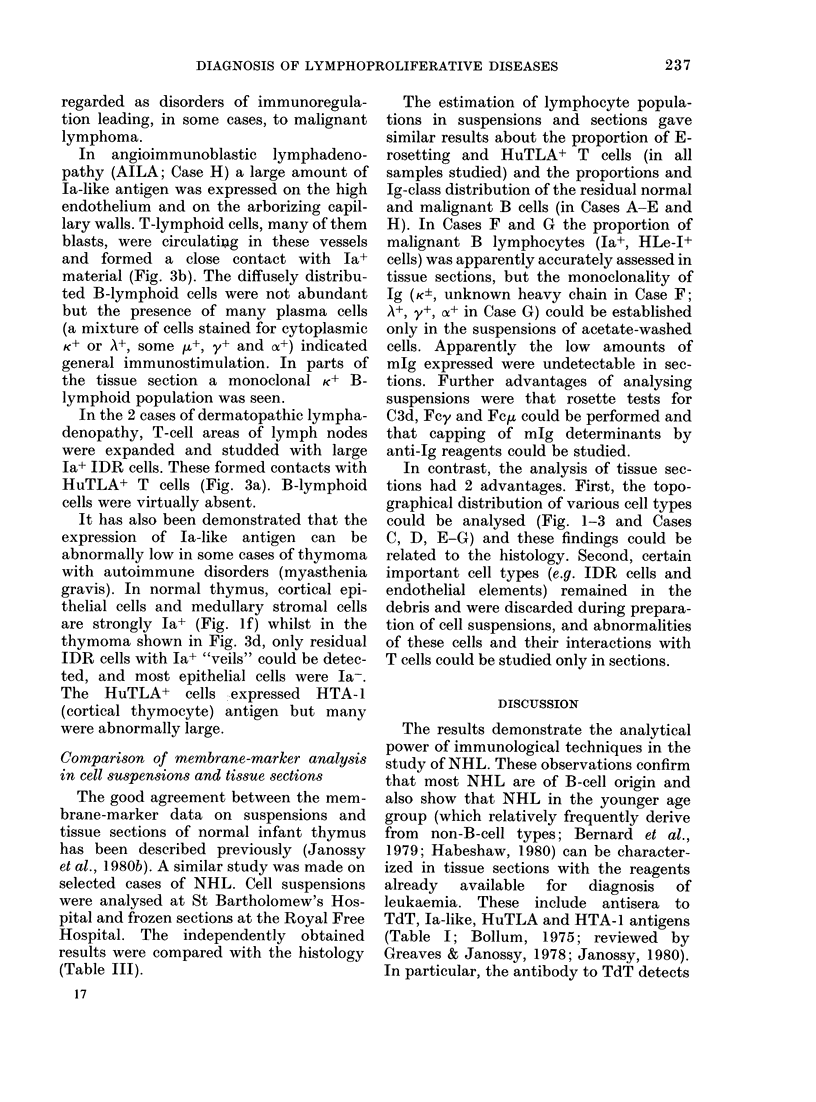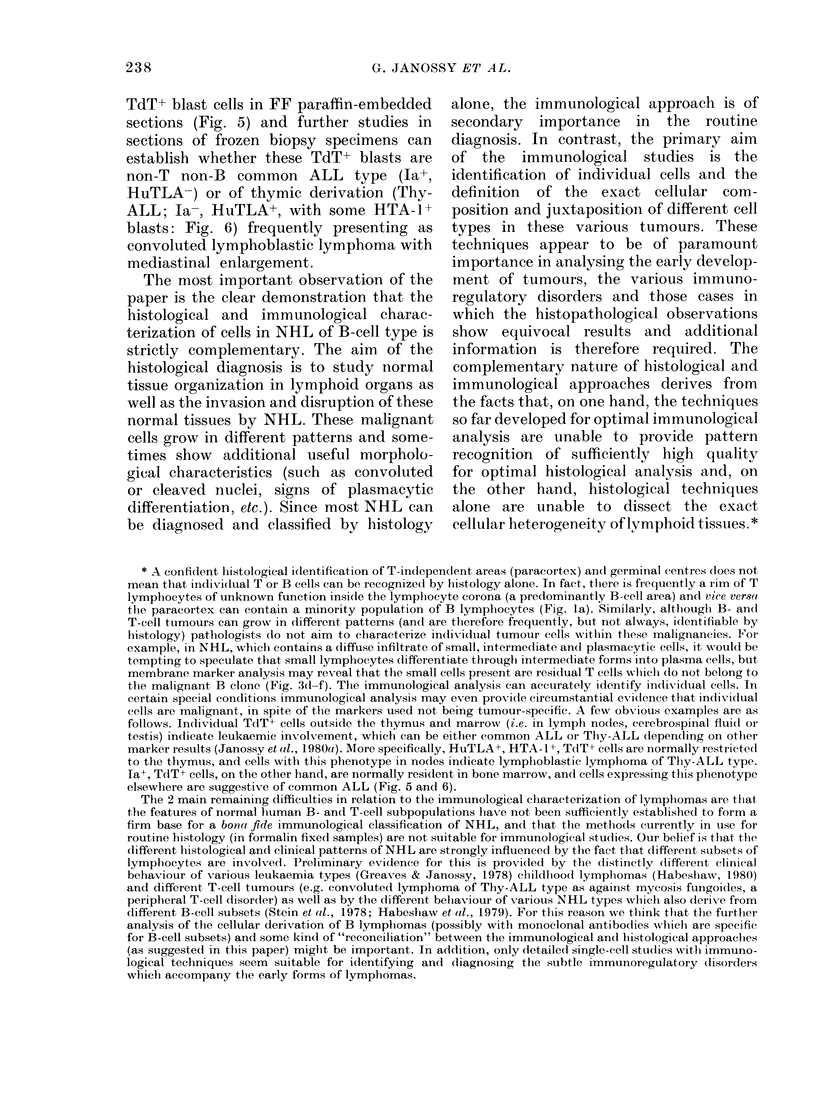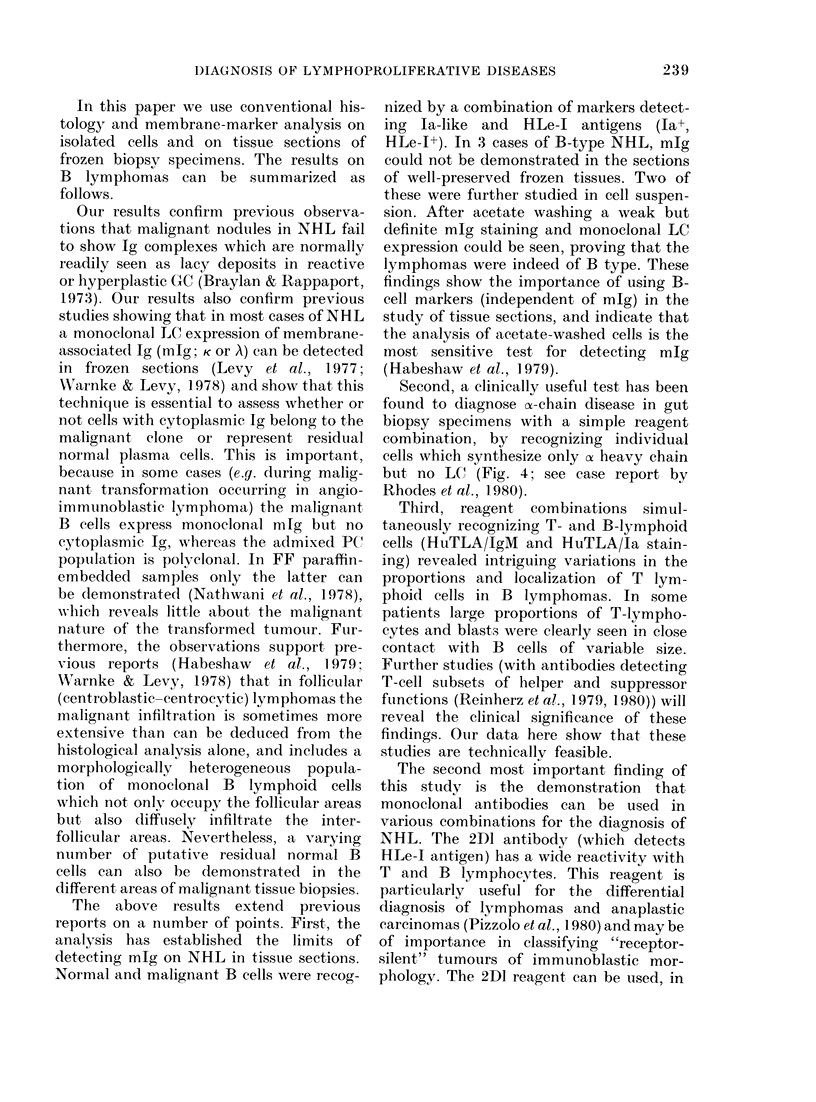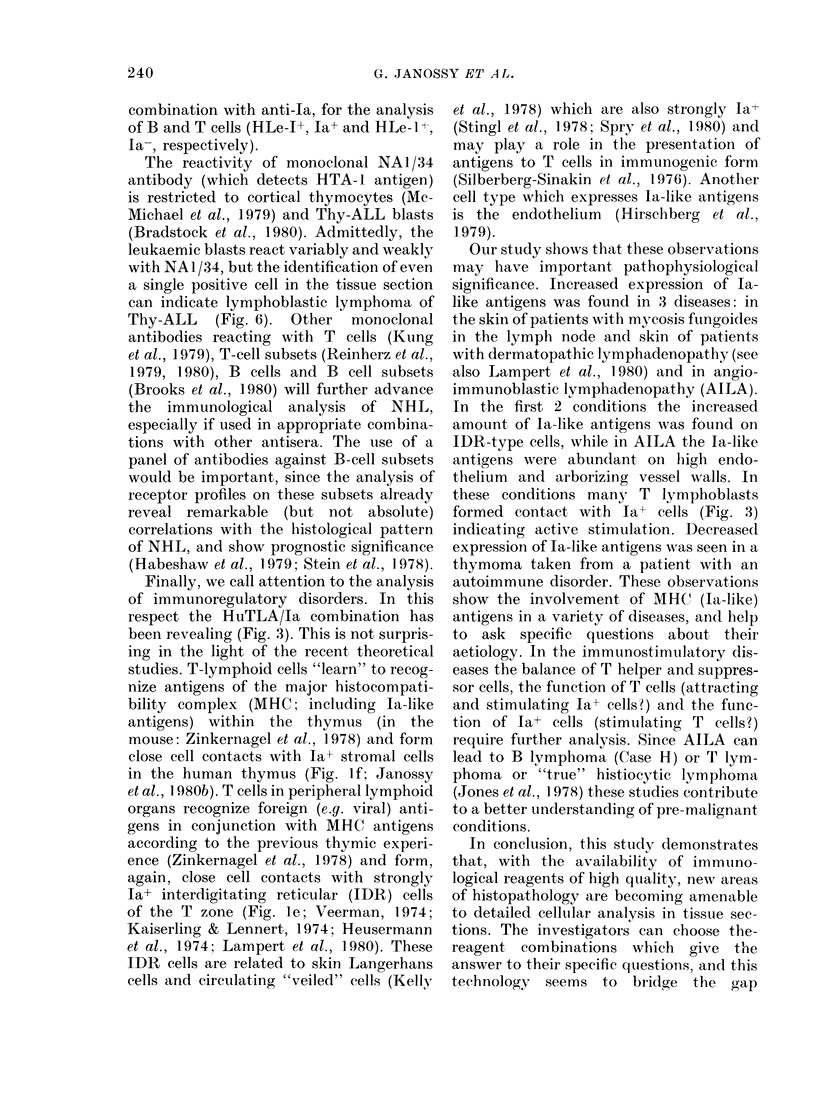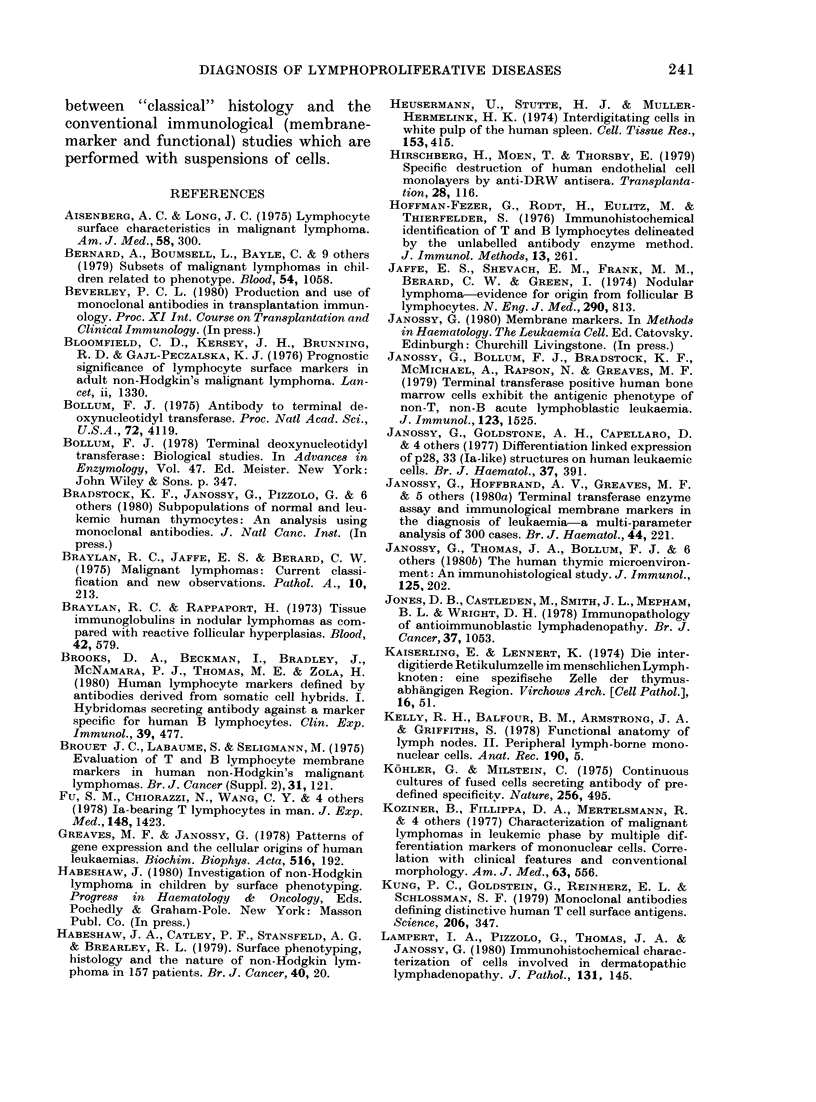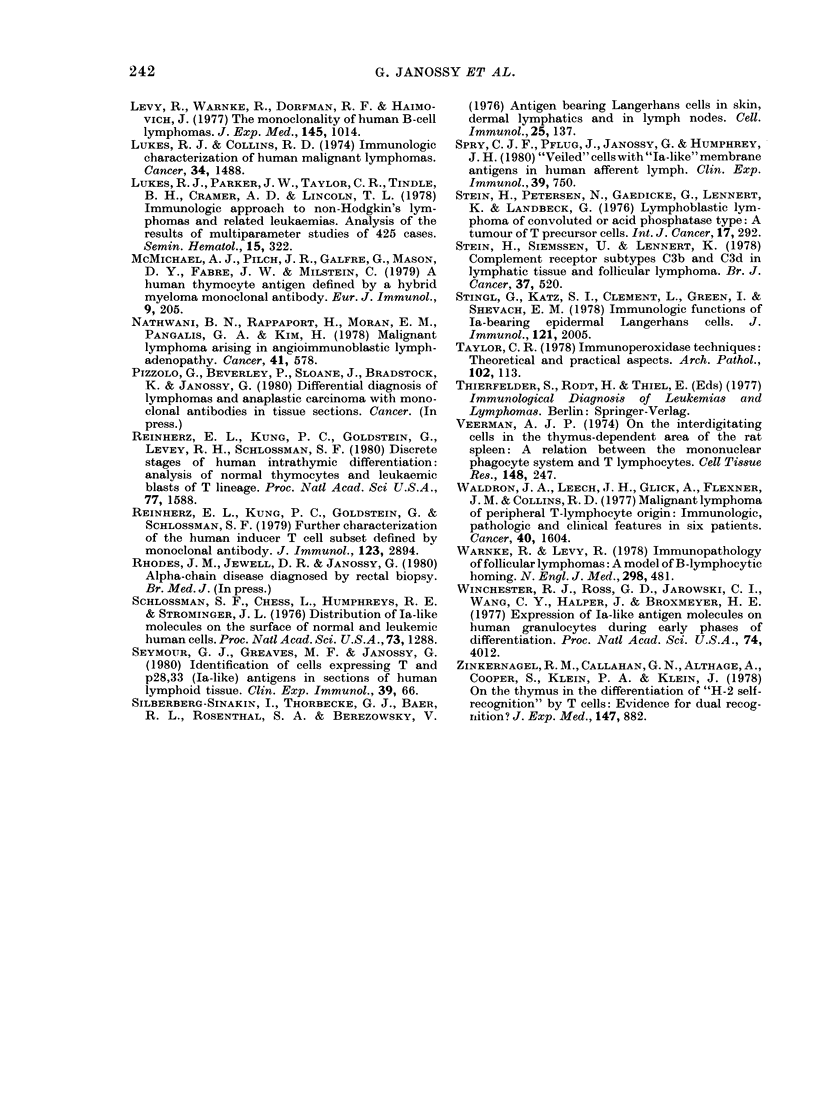# Immuno-histological diagnosis of lymphoproliferative diseases by selected combinations of antisera and monoclonal antibodies.

**DOI:** 10.1038/bjc.1980.222

**Published:** 1980-08

**Authors:** G. Janossy, J. A. Thomas, G. Pizzolo, S. M. Granger, J. McLaughlin, J. A. Habeshaw, A. G. Stansfeld, J. Sloane

## Abstract

**Images:**


*Br. J. Cancer* (1980) **42**, 224

### IMMUNO-HISTOLOGICAL DIAGNOSIS OF LYMPHOPROLIFERATIVE DISEASES BY SELECTED COMBINATIONS OF ANTISERA AND MONOCLONAL ANTIBODIES

G. JANOSSY*, J. A. THOMAS*, G. PIZZOLO*, S. M. GRANGER*, J. McLAUGHLIN*, J. A. HABESHAW†, A. G. STANSFELD AND J. SLOANE‡

*From the *Departments of Immunology and Histopathology, Royal Free Hospital, the †ICRF Medical Oncology Group and Department of Pathology, St Bartholomew's Hospital, London, and the ‡Department of Pathology, Royal Marsden Hospital, Sutton, Surrey*

Received 11 March 1980 Accepted 28 April 1980

**Summary.—Tissue sections of frozen biopsy specimens obtained from normal and hyperplastic human lymphoid tissues, 33 cases of non-Hodgkin lymphomas as well as various forms of immunoregulatory disorders (angioimmunoblastic and dermatopathic lymphadenopathy) were analysed in immunofluorescence tests (using red TRITC and green FITC double-labelling). A panel of antisera including well-characterized conventional reagents to immunoglobulin classes, T lymphoid and Ia-like antigens, and monoclonal antibodies was used. In selected cases the results were compared with the observations of membrane-marker staining on viable cells in suspension. The findings show that the immunological methods can give a very accurate analysis of the normal and malignant lymphoid cells, and can provide complementary information to conventional histology. The investigator can choose the reagent combinations which give answers to various specific questions: *e.g.* antisera to light chains establish the monoclonality of lymphomas, whilst staining combinations for human T and Ia-like antigens are particularly useful in various immunoregulatory disorders. Monoclonal antibodies will be particularly useful reagents for analysing the tissue distribution of lymphoid subpopulations and ancillary cells in tissue biopsy specimens.**

DURING RECENT YEARS non-Hodgkin lymphomas (NHL) have been characterized by immunological methods in a number of laboratories (Aisenberg & Long, 1975; Bloomfield *et al.*, 1976; Braylan *et al.*, 1975; Brouet *et al.*, 1975; Lukes *et al.*, 1978; Stein *et al.*, 1978; Habeshaw *et al.*, 1979). Staining of lymphocyte membrane antigens (with antisera) in formalin-fixed paraffin-embedded tissues has yielded variable results (Braylan & Rappaport, 1973; Taylor, 1978). Thus most of the published studies have been carried out on suspensions of cells obtained from teased lymph nodes, a technique which is better suited to the requirements of membrane-marker analysis. These studies have demonstrated that most lymphomas are of B-lymphoid origin and express "monoclonal" surface-membrane immunoglobulin (SmIg; with $\kappa$ or $\lambda$ light chain) and that complement receptors, Fc receptors and the capping characteristics of SmIg distinguish histologically distinct types of B lymphomas (Jaffe *et al.*, 1974; Stein *et al.*, 1978; Habeshaw *et al.*, 1979). A small proportion of lymphomas have been shown to be of T-cell origin (Lukes & Collins, 1974; Bloomfield *et al.*, 1976; Koziner *et al.*, 1977; Stein *et al.*, 1976; Waldron *et al.*, 1977).

An alternative possibility is to analyse the cellular composition of lymphomas by immunofluorescence (IF) in sections of

Address for reprints: Dr G. Janossy, Department of Immunology, Royal Free Hospital, Pond Street, Hampstead, London NW3 2QG, U.K.

frozen tissue. Recently, 3 factors have improved this technology. First, interchangeable filter sets used on the modern microscopes facilitate the simultaneous application of antibodies labelled with fluorescein–isothiocyanate (FITC–green) and tetraethylrhodamine–isothiocyanate (TRITC–red). Second, specific antisera and purified antibodies (eluted from immunoabsorbent columns) have been introduced for the immunodiagnosis of leukaemia (Thierfelder *et al.*, 1977; Bollum, 1975; Greaves & Janossy, 1978) and can also be applied for lymphoma diagnosis. Third, the production of "monoclonal" antibodies by somatic-cell hybridization (Köhler & Milstein, 1975) has now been established as an ideal method for preparing standard reagents which show low or undetectable nonspecific background staining in tissue sections (Pizzolo *et al.*, 1980; Janossy *et al.*, 1980*b*).

Previous investigators have analysed the "monoclonality" of Ig ($\kappa$ or $\lambda$ light chain) expression of NHL in tissue sections with antisera to $\kappa$ or $\lambda$ labelled with different fluorochromes (Levy *et al.*, 1977; Warnke & Levy, 1978). Other selected combinations of antibodies may also be important for the recognition of pathological lymphnode architecture in NHL and in the various forms of immunoregulatory disorders. In this paper we summarize the potential value of this approach.

### PATIENTS, MATERIALS AND METHODS

### *Patients*

The non-malignant tissues used were tonsils, adenoids, infant thymus tissue (removed during cardiac surgery) skin and gut biopsy samples. Lymphnode and skin biopsy specimens were analysed from a variety of diseases, including lymphoid hyperplasia and immunoregulatory disorders (18 cases) and suspected or diagnosed NHL (33 cases). These were classified according to the Rappaport and Kiel schemes (Table II).

### MATERIALS AND METHODS

Surgical biopsy specimens were divided; one part was fixed in 10% formol saline and processed for paraffin embedding, the second part was embedded in Ames OCT Compound (Miles Lab), frozen in liquid $N_2$ within 3 h of excision and stored at −70°C until sectioning. Five $\mu$m sections were air-dried and fixed in ethanol (5 min, 4°C) on glass slides and kept in phosphate-buffered saline (PBS+0·5% Na azide). From the third part of selected samples cell suspensions were made by teasing with blunt forceps and washed in medium.

### *Immunological studies in frozen tissue sections*

Excess fluid was removed from the slides. Antisera (see below) were layered on sections. The preparations were incubated at 20°C in a humid chamber for 30 min and washed in PBS for 30 min. When appropriate, conjugated second layers of antisera were added similarly. After washing, sections were mounted in buffered glycerol and examined under a Standard 14 Zeiss microscope equipped with a ×40 Phase oil objective and IV/F epifluorescence condenser containing selective filters (FITC) and rhodamine (TRITC). The following indirect IF test systems were used.

*Rabbit antiserum reacting with human T cells and thymocytes* (*R-anti-HuTLA*).—This was made against monkey thymocytes (Janossy *et al.*, 1977). The serum reacts with thymocytes and T lymphocytes; it also reacts with malignant cells in acute lymphoid leukaemia of thymic origin (Thy-ALL; Janossy *et al.*, 1980*a*) T-CLL, Sezary syndrome and mycosis fungoides. The serum is unreactive with normal B-lymphoid, myeloid and epithelial cells, and with non-T malignant cells: common ALL, acute myeloid leukaemia (AML) or B chronic lymphoid leukaemia (B-CLL). The serum was used at 1:10 dilution with a second layer goat anti-rabbit IgG coupled to FITC (G-anti-R-FITC; F/P=1:3). This was eluted from a rabbit IgG immunoabsorbent column and used at a concentration of 0·2 $\mu$g/ml.

*Chicken antiserum to Ia-like antigens C-anti-Ia*).—This was made against purified human p28,33 antigens (Janossy *et al.*, 1979). Anti-Ia-like sera react strongly with B lymphocytes, normal myeloblasts, some monocytes, tissue macrophages and interdigitating reticular cells, and weakly with a small subpopulation of T lymphocytes (Schlossman *et al.*, 1976; Winchester *et al.*, 1977; Janossy *et al.*, 1977, 1980*b*; Fu *et al.*, 1978). It is unreactive with thymocytes, most T lymphocytes, mature myeloid cells, platelets and erythrocytes. In malignant-cell populations, anti-Ia-like serum reacts with cells of B lymphoproliferative diseases, non-T non-B ALL and some AML but fails to react with Thy-ALL, most T lymphomas, T-CLL and Sezary syndrome (Janossy *et al.*, 1977; 1980*a*; Schlossman *et al.*, 1976; Winchester *et al.*, 1977). C-anti-Ia serum was used at 1:40 dilution on sections. The second layer was a sheep anti-chicken IgG coupled to TRITC or FITC (S-anti-C-TRITC or FITC) used at 1:10 dilution.

*Rabbit antiserum to calf terminal deoxynucleotidyl transferase* (*R-anti-TdT*).—This was eluted from TdT immunoabsorbent column and used at 0·5 $\mu$g/ml (Bollum, 1975). The reagent reacts with nuclear TdT in common ALL and Thy-ALL, in normal cortical thymocytes and 0·5–3% non-T non-B cells in normal marrow. Other cells are unreactive (Bollum, 1978; Janossy *et al.*, 1979). The reagent was used in dewaxed formalin-fixed paraffin-embedded sections (Thomas *et al.*, in preparation).

Two monoclonal antibodies were used in this study.

*Anti-human leucocyte antibody.*—This 2Dl antibody reacted with HLe-I antigen (Beverley, 1980); it had strong reactivity against lymphoid cells and lymphomas (Table I) and weak reactivity against myeloid cells, some macrophages and monocytes. It was unreactive with other tissues and non-lymphoid malignancies (Pizzolo *et al.*, 1980). The 2Dl antibody (a culture supernatant) was used at a dilution of 1:2.

*Anti-human thymocyte antibody.*—This was Nal/34 antibody reacting with HTA-1 antigen; it strongly reacted with cortical thymocyte, variably reacted with thymic ALL (Thy-ALL) and failed to react with all other cell types tested (McMichael *et al.*, 1979; Bradstock *et al.*, 1980). The antibody, a peritoneal exudate, was used diluted 1:20. At this high concentration the antibody saturates the binding sites but gives no non-specific staining.

Both markers were detected by goat anti-mouse IgG coupled to FITC or TRITC (G-anti-M-FITC or TRITC; first purchased from Nordic and later eluted from mouse IgG immunoadsorbent column) used at 0·5 $\mu$g/ml.

The following direct IF tests were used.

*Goat antiserum to human Ig* ($\kappa$, $\lambda$, $\mu$, $\gamma$) *coupled to FITC.*—G-anti-Hu-Ig-FITC (Behringwerke) was used at 1:40 dilution. Goat antiserum to human IgM ($\mu$-specific) coupled to TRITC or to FITC (G-anti-Hu-IgM reagents) were absorbed on light-chain, IgG and IgA immunoabsorbent columns and specifically eluted from IgM immunoadsorbent columns. Goat antiserum to human IgG ($\gamma$-specific) coupled to TRITC (G-anti-Hu-IgG-TRITC) was similarly processed, except that IgM absorbent was used and the pure antibodies were eluted from insoluble IgG. These were used at 0·2–0·5 $\mu$g/$\mu$l.

*Rabbit antiserum to human IgA* ($\alpha$-*specific*) *coupled to TRITC.*—R-anti-Hu-IgA; (Capel Labs) was absorbed on goat serum immunoabsorbent before use at 1:20. Selected rabbit antisera to $\kappa$ and $\lambda$ light chain were coupled to TRITC and FITC respectively (R-anti-$\kappa$-TRITC and R-anti-$\lambda$-FITC) and were used at 1:10.

Some reagent combinations were routinely used to detect the following antigens:

1. HuTLA (FITC)—Ia-like (TRITC; Fig. 1 and 3)

2. HuTLA (FITC)—IgM (TRITC; detecting SmIg $\mu$ on most B cells; Fig. 1)

3. HLe-I (FITC)—Ia-like (TRITC);

4. Ig (whole Ig, $\kappa$, $\lambda$, $\gamma$ and $\mu$- FITC)—IgA ($\alpha$-chain specific-TRITC: Fig. 4)

5. IgM ($\mu$-specific-FITC)—IgG ($\gamma$-specific-TRITC; Fig. 1)

6. Kappa ($\kappa$-TRITC)—lambda ($\lambda$-FITC; Fig. 1 and 2)

The first 3 combinations give the proportion of T and B lymphoid cells. The second 3 analyse the heavy-chain class and monoclonality of B-lymphoid and plasmacytic populations. Staining for HTA-1 antigen and nuclear TDT was performed on specific indications (childhood lymphomas, testicular biopsy specimens and lymphoblastic lymphomas of acid-phosphatase-positive or convoluted type; Fig. 5).

### *Membrane markers studies in cell suspensions*

*E rosettes* (*a T-lymphocyte and thymocyte marker*).—These were formed by mixing $10^6$ leucocytes with $40 \times 10^6$ sheep erythrocytes (RBC). The mixture was incubated for 10 min at 37 °C, centrifuged and further incubated as a pellet for 2 h at 4 °C. The nucleated cells binding >3 erythrocytes (RBC) were counted as rosettes. C3d rosettes were performed with ox RBC coated with rabbit–anti-ox RBC

IgM antibodies; sensitized cells were treated with human R3 reagent and washed twice. Leucocytes and C3d RBC were rosetted as above (Habeshaw *et al.*, 1979). Fc$\gamma$ and Fc$\mu$ rosettes were carried out with ox RBC coated with rabbit anti-ox RBC IgG and IgM, respectively (Habeshaw *et al.*, 1979).

*Surface-Ig staining.*—This has been carried out after elution of nonspecific immune complexes or Ig from the cell surface by incubation of cells in acetate buffer (pH 5·5) for 15–30 min at 37°C, washing in medium and a further incubation of cells for 1 h at 37°C (Habeshaw *et al.*, 1979). $10^6$ washed cells were incubated with FITC-conjugated anti-immunoglobulin reagents (see above) for 30 min at 4°C and washed twice before counting. All reagents were centrifuged at 110,000 g for 1 h before use.

*Cytoplasmic Ig staining.*—This was performed on cytocentrifuge preparations fixed in ethanol. These smears were stained with appropriate dilution of FITC-coupled antisera against $\kappa$, $\lambda$, $\mu$, $\gamma$, $\alpha$ and $\delta$ chains, or with the reagent combinations described above as 4, 5 and 6.

*Immunological studies in formalin-fixed paraffin-embedded sections*

Sections (4 $\mu$m thick) were dewaxed. Nuclear terminal deoxynucleotidyl transferase (TdT) in formalin-fixed paraffin-embedded sections was demonstrated by immunoperoxidase PAP technique (Thomas *et al.*, in preparation). The reaction sequence was similar to the method described by Taylor (1978) except that sections were treated with 0·1% deoxyribonuclease I (Sigma, D4763) in 0·1M $MgCl_2$ (pH6·5) for 30 min before application of the primary rabbit anti-TdT antibody (see above). The incubation with this antibody was continued at room temperature for 4–6 h. The subsequent antisera for PAP staining, however, only required 30 min incubation.

### RESULTS

*Preliminary experiments*

The reactivity of anti-HuTLA, anti-Ia, anti-$\mu$ antisera and the 2Dl antibody (detecting HLe-I antigen) was tested in formalin-fixed (FF) paraffin-embedded sections (using indirect IF with FITC and the PAP method) and on frozen sections (using indirect IF) of tonsil and infant thymus. In the FF preparations no HuTLA staining and very weak of variable Ia, HLe-I and membrane-associated $\mu$ staining was detected (although the cytoplasmic $\mu$ was clearly stained). The FITC stain gave slightly sharper resolution than PAP, but the intensity of staining above background was not satisfactory even when the 2Dl was used with affinity-purified second-layer antibody. In contrast, in frozen sections brilliant FITC membrane staining was obtained with all 4 reagents (Fig. 1). Similar results were reported in frozen sections with the PAP method by other groups (Hoffmann-Fezer *et al.*, 1976). Thus while these results do not exclude that appropriate modifications of fixation and/or embedding may yield acceptable staining (Seymour *et al.*, 1980) they indicate that conventional FF paraffin blocks are not suitable for membrane analysis, even with reagents of high quality.

It has also become clear that while the IF staining of frozen sections with any one of the reagents used was bright, the staining pattern was frequently difficult to interpret. Therefore combinations of reagents labelled with different fluorochromes (FITC and TRITC; Fig. 1) were used which gave easily interpretable results.

In contrast to membrane labelling it was feasible to stain nuclear TDT enzyme in FF paraffin-embedded blocks within the cortical areas of human thymus (Janossy *et al.*, 1980*b*). Both indirect IF and PAP labelling were successful (see below).

*Normal tissues*

Sections from frozen tissue biopsies were routinely studied with 6 reagent combinations (see METHODS). The observations on normal human thymus have been described previously (Janossy *et al.*, 1980*b*). The findings on tonsil and adenoids are summarized as follows.

The germinal centres (GC) contained a lacy network of apparently extracellular deposits strongly staining for $\mu$ (Fig. 1a)

TABLE I.—*Expression of various antigens on normal cells*

| Cells | HuTLA | HTA-1* | TdT† | Membrane Ig‡ | Cytoplasmic Ig | Ia | HLe-I* |
|---|---|---|---|---|---|---|---|
| Non-T, non-B precursor cell in marrow § | − | − | + + | − | − | + + | ± |
| Cortical thymocyte¶ | + + | + | + | − | − | − | + + |
| T lymphocyte | + +/+ | − | − | − | − | − | + + |
| B lymphocyte | − | − | − | + +/+ | +/− | + + | + + |
| Plasma cell | − | − | − | +/− | + + | ±/− | ± |
| Idr cells ‖ | − | − | − | − | − | + + + + | ± |
| Dendritic cell in germinal centre | − | − | − | − | − | + + + | ± |

* Monoclonal antibodies made by mouse B-cell–myeloma hybrids (McMichael *et al.*, 1979; Beverley *et al.*, 1980).

† Analysed in formalin-fixed paraffin-embedded samples by the PAP method (Fig. 6).

‡ Normal B-lymphocyte and plasma-cell populations consist of mixtures of $\kappa + \lambda$ cells (Fig. 1d).

§ The phenotype of this cell corresponds to non-T, non-B ALL and most non-T, non-B lymphomas in children (Janossy *et al.*, 1980*a*; Bernard *et al.*, 1979; Habeshaw, 1980).

¶ The phenotype of this cell is similar to thymic (Thy)-ALL and lymphoblastic lymphoma of convoluted type (see Fig. 5 for further details).

‖ Interdigitating reticular cells of T-cell zones (in lymph node, spleen, thymic medulla and thymic interlobular septae) and related cell types such as Langerhans cells of skin and circulating "veiled" cells (Lampert *et al.*, 1980; Spry *et al.*, 1980; Stingl *et al.*, 1978; Janossy *et al.*, 1980*b*).

and weakly staining for $\gamma$ (Fig. 1c) and $\alpha$. These areas were surrounded with a lymphocyte corona of variable width, which mostly consisted of B lymphocytes strongly staining for membrane-associated IgM ($\mu^+$) and negative for $\gamma$, $\alpha$ or T-lymphocyte marker (HuTLA$^-$; Fig. 1a and 1c). B cells in the corona were a heterogeneous (polyclonal) mixture of cells stained for either $\kappa$ or $\lambda$ light chain in a ratio of 2:1 (Fig. 1d). The distribution of $\kappa^+$ and $\lambda^+$ B cells in the corona was not totally random: in the plane of the section small clusters of 5–10 cells expressed identical light chains ($\kappa^+$ or $\lambda^+$). In the interfollicular paracortical areas T lymphocytes (HuTLA$^+$, $\mu^-$) dominated with 20–30% admixed, mostly $\mu^+$, B lymphocytes. The medullary cords contained a heterogeneous mixture of HuTLA$^+$ T cells, $\mu^+$ B cells and plasma cells (PC) with strong cytoplasmic Ig staining. The B-lymphoid and PC populations included $\mu^+$, $\gamma^+$ and $\alpha^+$ cells; in some tonsils and adenoids $\alpha^+$ PCs predominated. Again these were mixed $\kappa^+$ and $\lambda^+$ cells.

The HuTLA/Ia and the HLe-I/Ia combinations characterized the T and B lymphoid cells. Most T cells (HuTLA$^+$, HLe-I$^+$) were apparently Ia$^-$, although some HuTLA$^+$, Ia$^+$ larger T cells were also sometimes seen (Seymour *et al.*, 1980). B lymphocytes were HuTLA$^-$, HLe-I$^+$ with ring-like Ia$^+$ staining (Fig. 1b).

The proportion and tissue localization of B cells detected by $\mu$ staining (Fig. 1a) and by HLe-I/Ia staining were very similar, indicating that most B cells were recognized by both methods.

Two non-lymphoid cell types seemed to stain for Ia-like antigens: dendritic germinal-centre cells (Fig. 1b) and interdigitating reticular (IDR) cells in the T-cell areas (Fig. 1b & e). Of the 2 types, IDR cells showed the brighter Ia staining, and could be recognized by their protruding processes and "whiskers". Similar cells were seen in the paracortical areas of lymph nodes, periarteriolar region in spleen (Lampert *et al.*, 1980) and in the thymus medulla (Fig. 1f). Similarly, Langerhans cells in skin were also brightly Ia$^+$.

In addition to tonsils and adenoids, a number of reactive non-neoplastic samples from patients were also analysed. The diagnosis was as one of 2 groups: reactive follicular hyperplasia and reactive T-cell hyperplasia. In follicular hyperplasia (Fig. 1d; Case A in Table III) the

FIG. 1.—Immunofluorescence (IF) analysis of cryostat sections made from frozen tissue samples of normal human tonsil (a–e) and thymus (f). The sections were incubated with combinations of antisera labelled with different fluorochromes (FITC–green; TRIC–red). The same area was photographed with filters for FITC and TRIC as double exposure. (Membrane segments of adjacent cells stained with green and red, respectively, may appear yellow.) The antiserum combinations detected the following antigens: (a) HuTLA (FITC)—$\mu$ (TRIC): Ig complexes in the germinal centre (GC) are strongly $\mu^+$. Most lymphocytes in the lumphocyte corona and a few in the T-cell area carry membrane-associated $\mu$ (ring staining). In the few plasma cells the stain is cytoplasmic. (b) HuTLA (FITC)— Ia-like antigen (TRITC). Dendritic cells in the GC as well as B cells are Ia$^+$. Interdigitating reticular cells (idr) in the T area are also particularly strongly Ia$^+$ (see also e). (c) $\mu$ (FITC)—$\gamma$ (TRITC). Most B lymphocytes carry membrane $\mu$. Immune complexes in the GC contain mostly $\mu$ but some $\gamma$ is also present (greenish yellow). T cells show minimal staining. Three $\gamma^+$ plasma cells are seen. (d) light chains $\kappa$ (TRITC) and $\lambda$ (FITC). This area is the lymphocyte corona of a hyperplastic folliculus consisting of a mosaic of $\kappa^+$ and $\lambda^+$ cells (polyclonal B cells). Immune complexes are doubly stained (yellow), (e) HuTLA (FITC)—la-like antigen (TRITC). Tonsil section showing a T-dependent area with large numbers of interdigitating reticular cells (idr). (f) HTA-1 (FITC)—Ia-like antigen (TRITC) combination on normal infant thymus. C: cortex; M: medulla. The monoclonal antibody used (NA1/34) specifically reacts with HTA-1 antigen present only on cortical thymocytes (green) but absent on medullary thymic lymphocytes.

TABLE II.—*Expression of lymphoid markers in 33 cases of lymphoma*

| Patient | Histological diagnosis: Rappaport | Histological diagnosis: Kiel | Membrane markers*: HLe-I | Ia-like | Immunoglobulin: Heavy chain | Immunoglobulin: Light chain† | HuTLA | HTA-1 |
|---|---|---|---|---|---|---|---|---|
| 1 | Undifferentiated‡ | ML Lymphoblastic | +/± § | − | − | − | ++ | + |
| 2 | Diffuse histiocytic | ML Immunoblastic | +/± | − | − | − | ++ | + |
| 3–4 | Diffuse histiocytic | ML Centroblastic diffuse | +/± | − | − | − | ++ | − |
| 5 | Diffuse histiocytic | ML Centroblastic diffuse | +/± | − | − | − | ++ | − |
| 6–10 | Mycosis fungoides | | + | +++ (Idr) | − | − | ++/+ | |
| 11 | Well differentiated | ML Lymphocytic | + | ++ | μ | λ | − | |
| 12 | Well differentiated | ML Centrocytic | + | + | μ | κ | − | |
| 13 | Well differentiated (diffuse) | ML Centrocytic/ centroblastic | + | ++ | γ | λ | − | |
| 14 | Poorly differentiated Nodular | ML Centrocytic/ centroblastic follicular | + | ++ | μ | λ | − | |
| 15–16 | Poorly differentiated Nodular | ML Centrocytic/ centroblastic follicular | + | + | μ | κ | − | |
| 17–18 | Poorly differentiated Nodular | ML Centrocytic/ centroblastic follicular | + | ++ | γ | κ | − | |
| 19 | Poorly differentiated Nodular | ML Centrocytic/ centroblastic follicular | + | ++ | γ | λ | − | |
| 20 | Poorly differentiated Nodular/diffuse | ML Immunoblastic | + | ++ | not detectable | | − | |
| 21 | Poorly differentiated Nodular/diffuse | ML Centrocytic/ centroblastic Follicular/diffuse | ++ | ++ | μ | λ | − | |
| 22 | Poorly differentiated diffuse | ML Lymphoplasmacytic | + | ++ | μ | κ | − | |
| 23–24 | Poorly differentiated diffuse | ML Centrocytic diffuse | + | + | μ | κ | − | |
| 25 | Poorly differentiated diffuse | ML Centrocytic diffuse | + | + | μ | λ | − | |
| 26 | Mixed lymphocytic histiocytic nodular | ML Centrocytic/ centroblastic | + | + | γ | λ | − | |
| 27 | Mixed lymphocytic histiocytic nodular | ML Centrocytic/ centroblastic | + | ++ | not detectable | | − | |
| 28 | Mixed lymphocytic histiocyte diffuse | ML Centrocytic/ centroblastic | + + | ++ ++ | not detectable | | − | |
| 29 | Mixed lymphocytic histiocytic diffuse | Unclassifiable | + | ++ | μ | κ | − | |
| 30–31 | Diffuse histiocytic | ML Immunoblastic | + | ++ | α¶ | − | − | |
| 32 | Diffuse histiocytic | ML Immunoblastic | + | ++ | μ | κ | − | |
| 33 | Undifferentiated | ML Immunoblastic | + | ++ | − | κ‖ | − | |

* Membrane-like staining in tissue sections of frozen samples. In some cases the results were confirmed using cell suspensions prepared from the same tissue (see Table III).

† The dominant phenotype in the involved areas is shown: > 10/1 or < 1/5 κ/λ ratio.

‡ Patients 1–3 had thymic masses.

§ Variable expression on lymphoma cells: 20–25% of cells are +, the rest show weak (±) but definite staining.

¶ Only α-chain expression in tumour of ampulla of Vater and in α-chain disease.

‖ Only κ-chain expression.

expanded germinal centres contained large amounts of extracellular Ig deposits and a wide lymphocyte corona with polyclonal B cells (mixture of $\kappa^+$ and $\lambda^+$ cells). In reactive T-cell, hyperplasia (Case B in Table III) the paracortical areas contained mixtures of $HuTLA^+$, mostly $Ia^-$ T lymphoid cells and larger $HuTLA^-$, $Ia^+$ cells. Some of the latter population were round cells with no apparent long processes (histiocytes or macrophages?). The percentages of $Ig^+$ B cells were negligible.

FIG. 2.—Analysis of B lymphomas. Figures a, b, and c are from Case C shown in Table III. (a) Edge of the malignant follicle (mf) stained with Giemsa. (b) A similar area stained for HuTLA (FITC) and Ia-like antigen (TRITC). (c) Stained for $\kappa$ (TRITC) and $\lambda$ (FITC) indicating that both large follicular and smaller interfollicular cells belong to the same $\kappa^+$, $Ia^+$ B-cell clone. Note the few interspersed T-lymphoid cells of variable size in b and 2 residual normal $\lambda^+$ plasma cells in c.

Figures d, e and f are from Case E: (d) Giemsa stain. (e) Staining for HuTLA (FITC) and $\mu$ (TRICT). (f) Staining for $\kappa$ (TRITC) and $\lambda$ (FITC). Smaller cells are mostly T lymphocytes, whereas the intermediate to larger cells and the few plasmacytic cells with cytoplasmic Ig staining are $\kappa^+$, $\mu^+$ malignant cells. Insert in f shows a $\lambda^+$ lymphoma (Case D).

TABLE III.—*Case reports*

| | | |
|---|---|---|
| A | Histology: | Follicular hyperplasia. |
| | Suspension: | E: 35%; Ig: 43% (+ +/+, $\mu^+$, mixture of $\kappa^+$, $\lambda^+$); C3d: 36%; Fc$\gamma$: < 1%; Fc$\mu$: < 1%. |
| | Frozen section: | Germinal centres (GC) with Ig complex deposition. Cells in lymphocyte corona (ly c) are: Ig$^+$ (+ +, mixture of $\kappa^+$, $\lambda^+$). Paracortex: T cells. Few plasma cells (PC): mixture of $\kappa^+$, $\lambda^+$. HLe-I (monoclonal antibody): > 90% + +. For further details see Fig. 1. |
| B | Histology: | Expansion of paracortical area with lymphocytes and hyperplastic macrophages (tuberculosis?) |
| | Suspension: | E: 90%; some additional large cells. Ig: < 2%. |
| | Frozen section: | No GC, no Ig complexes. 60% T lymphoid cells (HuTLA$^+$, HLe-I$^+$, Ia$^-$) are admixed to 40% large Ia$^+$, Ig$^+$, HLe-I$^-$ cells (histiocytic cells, macrophages?). Ig: < 2%. |
| C | Histology: | Malignant lymphoma, centroblastic–centrocytic (Kiel), poorly differentiated nodular (Rappaport). |
| | Suspension: | E: 10%; Ig: 80% ($\kappa$: 80% + +; $\lambda$: 2% +; $\gamma$: 80% +/+; $\mu$: 3%; $\alpha$: < 1%); C3d: 30%; Fc$\gamma$: 3%; Fc$\mu$: < 1%; PC: 1–2% (mixture of $\kappa^+$, $\lambda^+$). |
| | Frozen section: | No GC, no Ig complexes. Nodular areas consist of large $\kappa^+$, $\gamma^+$ strongly Ia$^+$ cells; internodular areas consist of small $\kappa^+$, $\gamma^+$ moderately Ia$^+$ cells (Fig. 2a–c). 25% T lymphoid cells (some lymphocytic, some lymphoblastic) are diffusely distributed. Residual $\lambda^+$, $\mu^+$ population: 3%. PC: 1% (mixture of $\kappa^+$, $\lambda^+$). HLe-I: 90% + +. |
| D | Histology: | Malignant lymphoma, centroblastic–centrocytic (Kiel) follicular–diffuse? Poorly differentiated (Rappaport). |
| | Suspension: | E: 20–30%; Ig: 34% ($\lambda$: 50% + +; $\kappa$: < 2%; $\mu$: 30% + +; $\gamma$: 1–2% +; $\alpha$: < 1%); C3d: 40%; Fc$\gamma$: < 1%; Fc$\mu$: < 1%; PC: < 1% $\kappa^+$. |
| | Frozen section: | No GC, no Ig complexes. Large poorly defined areas: mostly medium to large cells (Ia$^+$, $\lambda^+$, $\mu^+$) admixed with 10–15% T lymphocytes and 2–3% $\kappa^+$ cells. Other areas: 30% medium to large cells ($\lambda^+$, $\mu^+$) admixed with 50% T lymphocytes and 20% small $\kappa^+$ cells (residual normal cells). PC: < 1%. HLe-I: 90% + +. |
| E | Histology: | Malignant lymphoma, lymphoplasmacytoid–polymorphic (Kiel), poorly differentiated diffuse (Rappaport). |
| | Suspension: | E: 23%; Ig: 45% ($\kappa$: 50% + +; $\lambda$: < 1%; $\mu$: 50% + +; $\gamma$: 5% ±; $\alpha$: < 1%); C3d: < 1%; Fc$\gamma$: 4%; Fc$\mu$: 2%; PC: 5% ($\kappa^+$, $\mu^+$, $\lambda^+$). |
| | Frozen section: | No GC, no Ig complexes. Diffuse infiltration: 60% heterogeneous medium to large lymphoid cells (Ia$^+$, $\kappa^+$, $\mu^+$). 5% plasmacytoid cells ($\kappa^+$, $\mu^+$); 35% are small T lymphocytes (Fig. 2d–f). Residual $\lambda^+$ population: < 1%. HLe-I: > 90% + +. |
| F | Histology: | Malignant lymphoma, immunoblastic with residual T-cell areas (Kiel), diffuse histiocytic (Rappaport). |
| | Suspension: | E: 38%; Ig: 60% ($\kappa$: 50% ±; $\lambda$: < 1%; $\mu$, $\gamma$, $\alpha$: not detectable; C3d: < 1%; Fc$\gamma$: < 1%; Fc$\mu$: < 1%; PC: < 1%. |
| | Frozen section: | No GC, no Ig complexes. Two different areas: *Area A:* > 90% large Ia$^+$, HLe-I$^+$, HuTLA$^-$ blast cells with no detectable membrane-associated Ig. *Area B:* (Fig. 3b) 85% T cells (HuTLA, HLe-I$^+$, Ia$^-$) in close contact with 15% interdigitating reticular cells (idr cells; Ia: very strongly positive, HLe-I$^\pm$, HuTLA$^-$). |
| G | Histology: | Malignant follicular lymphoma transforming into immunoblastic lymphoma (Kiel), poorly differentiated nodular (Rappaport). |
| | Suspension: | E: 10%; Ig: 65% ($\lambda$: 50%, weak but definite +; $\kappa$: 1%; $\gamma$: 50% ±; $\alpha$: 50%; $\mu$: 3% + +); C3d: < 1%; Fc$\gamma$: < 1%; Fc$\mu$: < 1%; PC: 4% (mixture of $\kappa^+$, $\lambda^+$). |
| | Frozen section: | No GC, no Ig complexes. Large, poorly defined areas with 70% large Ia$^+$, HLe-I$^+$ blast cells (membrane-associated Ig is undetectable) admixed with 20% T lymphocytes (HuTLA$^+$, HLe–I$^+$) 10% B cells (mixture of $\kappa^+$, $\lambda^+$) and 4% PC ($\kappa^+$, $\lambda^+$). |
| H | Histology: | Angioimmunoblastic lymphadenopathy. |
| | Suspension: | E: 60%; Ig: 23% ($\kappa$: 17% + +; $\lambda$: 4% + +; $\mu$: 14%; $\gamma$: 6%; $\alpha$: 2%). C3d: 10%; Fc$\gamma$: 4%; Fc$\mu$: 2%; PC: 6% (mixture of $\kappa^+$, $\lambda^+$). |
| | Frozen section: | No GC. (Diffuse deposition of Ig in some areas partially obscuring membrane-associated Ig staining). The HuTLA/Ia combination reveals diffuse infiltration with strongly Ia$^+$ high endothelium. "Whirls" of Ia$^+$ material correspond to arborizing endothelium (Fig. 3*b*). Small clump of $\kappa^+$ monoclonal B cells (malignant transformation?). Plasma cells: polyclonal (some $\kappa^+$, some $\lambda^+$). |
| I | Histology: | Lymphoblastic lymphoma, convoluted, acid-phosphatase-positive (Kiel), undifferentiated (Rappaport). |
| | Frozen section: | No GC. Diffuse extravasal exudate weakly staining for $\gamma$, $\kappa$ and $\lambda$. No membrane-associated staining with anti-Ig reagents. The blasts stain for HuTLA (+ +), HLe-I (weakly), a thymocyte-specific antigen (HTA-1; heterogeneous staining, many blasts are negative, Fig. 6a–b) and are Ia$^-$. |
| J | Histology: | Mycosis fungoides (skin biopsy; cell suspension unobtainable). |
| | Frozen section: | The HuTLA/Ia combination reveals localized infiltrates of T-lymphoid cells (HuTLA$^+$, Ia$^-$ or Ia$^\pm$) in the epidermis and dermis. Adjacent cells with extensive processes express large amounts of Ia (Fig. 4d). Ig: < 1%. |

FIG. 3.—The use of HuTLA (FITC)–Ia (TRITC) combination in the analysis of immunoregulatory and related disorders. (a) Lymphnode biopsy in dermatopathic lymphadenopathy with highly increased numbers of $Ia^+$ interdigitating (idr) cells and surrounding T lymphocytes. Blood vessel (bv) is also $Ia^+$. (b) Angioimmunoblastic lymphadenopathy with "whirls" of arborizing endothelium and T cells of variable sizes. Note that the high endothelium is strongly $Ia^+$ (Case H). (c) Mycosis fungoides with greatly increased numbers of $Ia^+$ cells in the dermis (Case J). In the lower part of the field T cells attach to the $Ia^+$ cells. Top: epidermis. (d) Thymoma showing residual idr-type cells ($Ia^+$ cells with veils). The $HuTLA^+$ cells are larger than normal small thymocytes. Many cells fail to stain with either antisera. *Cf.* Fig. 1f.

*B-cell non-Hodgkin lymphomas*

In the 23 B-lymphoma cases studied, the normal architecture was distorted: deposition of lacy Ig complexes and a well-demarcated lymphocyte corona were absent in all cases studied. The malignant B cells were $Ia^+$ $HLe\text{-}I^+$, and mIg staining was detected in 20/23 cases seen. In 18 cases the monoclonality of light-chain (LC) expression ($\kappa:\lambda$ ratios $>10:1$ or $<1:5$) was demonstrated (Fig. 3e and Table II). In some follicular (centroblastic–centrocytic) lymphomas the extent of malignant involvement was greater than suspected on the basis of histology (Fig. 2a–c). The immunological analysis indicated that a large proportion of smaller, moderately $Ia^+$ B lymphocytes in the interfollicular area expressed the light- and heavy-chain characteristics of the "monoclonal" B-lymphoma cells seen in the neoplastic follicules (Fig. 2c). The $\kappa/\lambda$ double-staining in frozen tissue sections was ideal to assess the degree of plasmacytic differentiation within the malignant clone. When the cytoplasmic Ig in plasma cells (PC) had the same predominant LC type as the mIg on B-lymphoma cells (*e.g.* Fig. 2f) the plasmacytic cells were judged to be part of the malignant clone. In contrast, in $\sim 25\%$ of cases $>3\%$ plasma cells expressed near-normal $\kappa/\lambda$ ratio and were, most probably, not malignant (*cf.* Case G in Table III and $\lambda^+$ cells in Fig. 2c). This was most obvious in angioimmunoblastic lymphadenopathy, where $\kappa^+$ B lymphocytes were detected in one part of the sample (malignant ?) whilst the mature PCs were a mixture of $\kappa^+$ and $\lambda^+$ cells (Case H).

FIG. 4.—Diagnosis of $\alpha$-chain disease in gut biopsy. Section of rectal biopsy specimen was stained with anti-$\alpha$ (heavy-chain specific TRITC) and with mixture of anti-$\kappa$ and anti-$\lambda$ light-chain-specific antisera (FITC). The same field was photographed with TRITC (a) and FITC (b) filters. Most cells synthesize $\alpha$ but no light chain (abnormal cells). Stars indicate plasma cells synthesizing the whole IgA molecule ($\alpha$+light chain). Arrows point to plasma cells synthesizing Ig other than IgA. When stained in adjacent section for $\kappa$ (TRITC) + $\lambda$ (TRITC) these were shown to be a residual mixed population of $\kappa^+$ and $\lambda^+$ normal plasma cells.

In 2 cases of gut-associated malignancies, cells stained for $\alpha$ chain but failed to stain for $\kappa$ or $\lambda$ chain. One case was a lymphoma involving the ampulla of Vater with membrane-associated $\alpha$-chain disease. The diagnosis was made with the Ig ($\kappa^+\lambda$/IgA $\alpha$chain-specific) staining combination on a rectal biopsy specimen. This discriminated between malignant ($\kappa^-\lambda^-/\alpha^+$) and residual normal plasma-cell populations ($\kappa^+$ or $\lambda^+$; Fig. 4). The diagnosis was confirmed 1 month later by immunochemical methods.

Malignant B cells in the various cases of NHL showed a different relationship to residual normal lymphoid cells. T lymphocytes were sometimes present in high proportions (Cases D–F). In a few diffuse lymphomas (*e.g.* Case E) many small lymphocytes were T cells whilst the B-lymphoid clone consisted of medium to large cells with plasmacytic differentiation (Fig. 2f). In other cases, mostly in centroblastic–centrocytic lymphomas, the HuTLA$^+$ T-lymphoid cells included blasts around the edge of the malignant follicles, as well as T lymphocytes in the interfollicular areas (Fig. 2b). Finally, in further cases relatively normal or hyperactive T lymphoid areas were observed to be separate from the malignant B lymphoid elements (Case D).

Taken together, the analysis of malignant and normal cells in sections described the cellular organization of the different classes of NHL. The follicular and inter-

FIG. 5.—Demonstration of nuclear terminal deoxynucleotidyl transferase (TdT) in lymphoid leukaemic blast cells infiltrating the testis. Rabbit anti-TdT antibody was labelled with the PAP method in paraffin-embedded, dewaxed and DNA ase-treated sections (a). Infiltrating acute lymphoblastic leukaemia cells are stained for nuclear TdT (arrows point to some of the TdT$^+$ cells). Second layer (PAP only) shows minimal staining (b). The fields were photographed with phase contrast to visualize unstained cells. (*) Seminiferous tubule.

FIG. 6.—Characterization of lymphoblastic lymphoma of thymic phenotype (Case I). (A) Staining for HuTLA, (B) staining for HTA-1 antigen. Inserts show the cortico-medullary junction of infant thymus stained with the same reagents. Cortical thymocytes are HuTLA$^+$, HTA-1$^+$. c: cortex; m: medulla.

follicular areas were distinguished (Cases C and D) and the peculiarities of tumours were described in cellular terms (Cases F–H). The results complemented the histological observations.

*Non-T, non-B ALL blasts and T lymphomas*

Fifteen to 25% of childhood lymphomas ("receptor-silent" tumours with lymphoblastic morphology) consist of non-T, non-B ALL blasts (Bernard *et al.*, 1979; Habeshaw, 1980) and develop leukaemia of the same type. We diagnosed these blasts in tissue sections from involved testicular biopsy specimens, and showed the HuTLA$^-$, mIg$^-$, Ia$^+$ phenotype. These blasts expressed nuclear TdT (Fig. 5).

Ten of the 33 NHL cases studied were T-lymphoid malignancies (HuTLA$^+$, Ia$^-$). Two of these were lymphoblastic lymphomas with a subset of blasts weakly expressing HTA-1, a cortical thymocyte antigen (Fig. 6) and HLe-I. This corresponds to the phenotype of thymic ALL (Table I and Bradstock *et al.*, 1980). Five cases of mycosis fungoides and 2 cases of T lymphoma had the phenotype of peripheral T cells (HuTLA$^+$, HLe-I$^+$, Ia$^-$, HTA-1$^-$). One of the latter was studied further and failed to express TdT (TdT$^-$).

An unexpected finding was the increased number of Ia$^+$ reticular cells (Langerhans cells?) in mycosis fungoides in the epidermis, as well as in the dermis (Fig. 3c). This indicates that mycosis fungoides is not an isolated disorder of the T-cell lineage, but involves other cell types (see below).

*Immunoregulatory disorders*

The HuTLA/Ia combination revealed abnormalities in conditions which can be regarded as disorders of immunoregulation leading, in some cases, to malignant lymphoma.

In angioimmunoblastic lymphadenopathy (AILA; Case H) a large amount of Ia-like antigen was expressed on the high endothelium and on the arborizing capillary walls. T-lymphoid cells, many of them blasts, were circulating in these vessels and formed a close contact with $Ia^+$ material (Fig. 3b). The diffusely distributed B-lymphoid cells were not abundant but the presence of many plasma cells (a mixture of cells stained for cytoplasmic $\kappa^+$ or $\lambda^+$, some $\mu^+$, $\gamma^+$ and $\alpha^+$) indicated general immunostimulation. In parts of the tissue section a monoclonal $\kappa^+$ B-lymphoid population was seen.

In the 2 cases of dermatopathic lymphadenopathy, T-cell areas of lymph nodes were expanded and studded with large $Ia^+$ IDR cells. These formed contacts with $HuTLA^+$ T cells (Fig. 3a). B-lymphoid cells were virtually absent.

It has also been demonstrated that the expression of Ia-like antigen can be abnormally low in some cases of thymoma with autoimmune disorders (myasthenia gravis). In normal thymus, cortical epithelial cells and medullary stromal cells are strongly $Ia^+$ (Fig. 1f) whilst in the thymoma shown in Fig. 3d, only residual IDR cells with $Ia^+$ "veils" could be detected, and most epithelial cells were $Ia^-$. The $HuTLA^+$ cells expressed HTA-1 (cortical thymocyte) antigen but many were abnormally large.

*Comparison of membrane-marker analysis in cell suspensions and tissue sections*

The good agreement between the membrane-marker data on suspensions and tissue sections of normal infant thymus has been described previously (Janossy *et al.*, 1980*b*). A similar study was made on selected cases of NHL. Cell suspensions were analysed at St Bartholomew's Hospital and frozen sections at the Royal Free Hospital. The independently obtained results were compared with the histology (Table III).

The estimation of lymphocyte populations in suspensions and sections gave similar results about the proportion of E-rosetting and $HuTLA^+$ T cells (in all samples studied) and the proportions and Ig-class distribution of the residual normal and malignant B cells (in Cases A–E and H). In Cases F and G the proportion of malignant B lymphocytes ($Ia^+$, $HLe\text{-}I^+$ cells) was apparently accurately assessed in tissue sections, but the monoclonality of Ig ($\kappa^\pm$, unknown heavy chain in Case F; $\lambda^+$, $\gamma^+$, $\alpha^+$ in Case G) could be established only in the suspensions of acetate-washed cells. Apparently the low amounts of mIg expressed were undetectable in sections. Further advantages of analysing suspensions were that rosette tests for C3d, $Fc\gamma$ and $Fc\mu$ could be performed and that capping of mIg determinants by anti-Ig reagents could be studied.

In contrast, the analysis of tissue sections had 2 advantages. First, the topographical distribution of various cell types could be analysed (Fig. 1–3 and Cases C, D, E–G) and these findings could be related to the histology. Second, certain important cell types (*e.g.* IDR cells and endothelial elements) remained in the debris and were discarded during preparation of cell suspensions, and abnormalities of these cells and their interactions with T cells could be studied only in sections.

### DISCUSSION

The results demonstrate the analytical power of immunological techniques in the study of NHL. These observations confirm that most NHL are of B-cell origin and also show that NHL in the younger age group (which relatively frequently derive from non-B-cell types; Bernard *et al.*, 1979; Habeshaw, 1980) can be characterized in tissue sections with the reagents already available for diagnosis of leukaemia. These include antisera to TdT, Ia-like, HuTLA and HTA-1 antigens (Table I; Bollum, 1975; reviewed by Greaves & Janossy, 1978; Janossy, 1980). In particular, the antibody to TdT detects

TdT$^+$ blast cells in FF paraffin-embedded sections (Fig. 5) and further studies in sections of frozen biopsy specimens can establish whether these TdT$^+$ blasts are non-T non-B common ALL type (Ia$^+$, HuTLA$^-$) or of thymic derivation (Thy-ALL; Ia$^-$, HuTLA$^+$, with some HTA-1$^+$ blasts: Fig. 6) frequently presenting as convoluted lymphoblastic lymphoma with mediastinal enlargement.

The most important observation of the paper is the clear demonstration that the histological and immunological characterization of cells in NHL of B-cell type is strictly complementary. The aim of the histological diagnosis is to study normal tissue organization in lymphoid organs as well as the invasion and disruption of these normal tissues by NHL. These malignant cells grow in different patterns and sometimes show additional useful morphological characteristics (such as convoluted or cleaved nuclei, signs of plasmacytic differentiation, *etc.*). Since most NHL can be diagnosed and classified by histology alone, the immunological approach is of secondary importance in the routine diagnosis. In contrast, the primary aim of the immunological studies is the identification of individual cells and the definition of the exact cellular composition and juxtaposition of different cell types in these various tumours. These techniques appear to be of paramount importance in analysing the early development of tumours, the various immunoregulatory disorders and those cases in which the histopathological observations show equivocal results and additional information is therefore required. The complementary nature of histological and immunological approaches derives from the facts that, on one hand, the techniques so far developed for optimal immunological analysis are unable to provide pattern recognition of sufficiently high quality for optimal histological analysis and, on the other hand, histological techniques alone are unable to dissect the exact cellular heterogeneity of lymphoid tissues.*

* A confident histological identification of T-independent areas (paracortex) and germinal centres does not mean that individual T or B cells can be recognized by histology alone. In fact, there is frequently a rim of T lymphocytes of unknown function inside the lymphocyte corona (a predominantly B-cell area) and *vice versa* the paracortex can contain a minority population of B lymphocytes (Fig. 1a). Similarly, although B- and T-cell tumours can grow in different patterns (and are therefore frequently, but not always, identifiable by histology) pathologists do not aim to characterize individual tumour cells within these malignancies. For example, in NHL, which contains a diffuse infiltrate of small, intermediate and plasmacytic cells, it would be tempting to speculate that small lymphocytes differentiate through intermediate forms into plasma cells, but membrane marker analysis may reveal that the small cells present are residual T cells which do not belong to the malignant B clone (Fig. 3d–f). The immunological analysis can accurately identify individual cells. In certain special conditions immunological analysis may even provide circumstantial evidence that individual cells are malignant, in spite of the markers used not being tumour-specific. A few obvious examples are as follows. Individual TdT$^+$ cells outside the thymus and marrow (*i.e.* in lymph nodes, cerebrospinal fluid or testis) indicate leukaemic involvement, which can be either common ALL or Thy-ALL depending on other marker results (Janossy *et al.*, 1980*a*). More specifically, HuTLA$^+$, HTA-1$^+$, TdT$^+$ cells are normally restricted to the thymus, and cells with this phenotype in nodes indicate lymphoblastic lymphoma of Thy-ALL type. Ia$^+$, TdT$^+$ cells, on the other hand, are normally resident in bone marrow, and cells expressing this phenotype elsewhere are suggestive of common ALL (Fig. 5 and 6).

The 2 main remaining difficulties in relation to the immunological characterization of lymphomas are that the features of normal human B- and T-cell subpopulations have not been sufficiently established to form a firm base for a *bona fide* immunological classification of NHL, and that the methods currently in use for routine histology (in formalin fixed samples) are not suitable for immunological studies. Our belief is that the different histological and clinical patterns of NHL are strongly influenced by the fact that different subsets of lymphocytes are involved. Preliminary evidence for this is provided by the distinctly different clinical behaviour of various leukaemia types (Greaves & Janossy, 1978) childhood lymphomas (Habeshaw, 1980) and different T-cell tumours (e.g. convoluted lymphoma of Thy-ALL type as against mycosis fungoides, a peripheral T-cell disorder) as well as by the different behaviour of various NHL types which also derive from different B-cell subsets (Stein *et al.*, 1978; Habeshaw *et al.*, 1979). For this reason we think that the further analysis of the cellular derivation of B lymphomas (possibly with monoclonal antibodies which are specific for B-cell subsets) and some kind of "reconciliation" between the immunological and histological approaches (as suggested in this paper) might be important. In addition, only detailed single-cell studies with immunological techniques seem suitable for identifying and diagnosing the subtle immunoregulatory disorders which accompany the early forms of lymphomas.

In this paper we use conventional histology and membrane-marker analysis on isolated cells and on tissue sections of frozen biopsy specimens. The results on B lymphomas can be summarized as follows.

Our results confirm previous observations that malignant nodules in NHL fail to show Ig complexes which are normally readily seen as lacy deposits in reactive or hyperplastic GC (Braylan & Rappaport, 1973). Our results also confirm previous studies showing that in most cases of NHL a monoclonal LC expression of membrane-associated Ig (mIg; $\kappa$ or $\lambda$) can be detected in frozen sections (Levy *et al.*, 1977; Warnke & Levy, 1978) and show that this technique is essential to assess whether or not cells with cytoplasmic Ig belong to the malignant clone or represent residual normal plasma cells. This is important, because in some cases (*e.g.* during malignant transformation occurring in angio-immunoblastic lymphoma) the malignant B cells express monoclonal mIg but no cytoplasmic Ig, whereas the admixed PC population is polyclonal. In FF paraffin-embedded samples only the latter can be demonstrated (Nathwani *et al.*, 1978), which reveals little about the malignant nature of the transformed tumour. Furthermore, the observations support previous reports (Habeshaw *et al.*, 1979; Warnke & Levy, 1978) that in follicular (centroblastic–centrocytic) lymphomas the malignant infiltration is sometimes more extensive than can be deduced from the histological analysis alone, and includes a morphologically heterogeneous population of monoclonal B lymphoid cells which not only occupy the follicular areas but also diffusely infiltrate the interfollicular areas. Nevertheless, a varying number of putative residual normal B cells can also be demonstrated in the different areas of malignant tissue biopsies.

The above results extend previous reports on a number of points. First, the analysis has established the limits of detecting mIg on NHL in tissue sections. Normal and malignant B cells were recognized by a combination of markers detecting Ia-like and HLe-I antigens (Ia$^+$, HLe-I$^+$). In 3 cases of B-type NHL, mIg could not be demonstrated in the sections of well-preserved frozen tissues. Two of these were further studied in cell suspension. After acetate washing a weak but definite mIg staining and monoclonal LC expression could be seen, proving that the lymphomas were indeed of B type. These findings show the importance of using B-cell markers (independent of mIg) in the study of tissue sections, and indicate that the analysis of acetate-washed cells is the most sensitive test for detecting mIg (Habeshaw *et al.*, 1979).

Second, a clinically useful test has been found to diagnose $\alpha$-chain disease in gut biopsy specimens with a simple reagent combination, by recognizing individual cells which synthesize only $\alpha$ heavy chain but no LC (Fig. 4; see case report by Rhodes *et al.*, 1980).

Third, reagent combinations simultaneously recognizing T- and B-lymphoid cells (HuTLA/IgM and HuTLA/Ia staining) revealed intriguing variations in the proportions and localization of T lymphoid cells in B lymphomas. In some patients large proportions of T-lymphocytes and blasts were clearly seen in close contact with B cells of variable size. Further studies (with antibodies detecting T-cell subsets of helper and suppressor functions (Reinherz *et al.*, 1979, 1980)) will reveal the clinical significance of these findings. Our data here show that these studies are technically feasible.

The second most important finding of this study is the demonstration that monoclonal antibodies can be used in various combinations for the diagnosis of NHL. The 2Dl antibody (which detects HLe-I antigen) has a wide reactivity with T and B lymphocytes. This reagent is particularly useful for the differential diagnosis of lymphomas and anaplastic carcinomas (Pizzolo *et al.*, 1980) and may be of importance in classifying "receptor-silent" tumours of immunoblastic morphology. The 2Dl reagent can be used, in combination with anti-Ia, for the analysis of B and T cells (HLe-I$^+$, Ia$^+$ and HLe-1$^+$, Ia$^-$, respectively).

The reactivity of monoclonal NA1/34 antibody (which detects HTA-1 antigen) is restricted to cortical thymocytes (McMichael *et al.*, 1979) and Thy-ALL blasts (Bradstock *et al.*, 1980). Admittedly, the leukaemic blasts react variably and weakly with NA1/34, but the identification of even a single positive cell in the tissue section can indicate lymphoblastic lymphoma of Thy-ALL (Fig. 6). Other monoclonal antibodies reacting with T cells (Kung *et al.*, 1979), T-cell subsets (Reinherz *et al.*, 1979, 1980), B cells and B cell subsets (Brooks *et al.*, 1980) will further advance the immunological analysis of NHL, especially if used in appropriate combinations with other antisera. The use of a panel of antibodies against B-cell subsets would be important, since the analysis of receptor profiles on these subsets already reveal remarkable (but not absolute) correlations with the histological pattern of NHL, and show prognostic significance (Habeshaw *et al.*, 1979; Stein *et al.*, 1978).

Finally, we call attention to the analysis of immunoregulatory disorders. In this respect the HuTLA/Ia combination has been revealing (Fig. 3). This is not surprising in the light of the recent theoretical studies. T-lymphoid cells "learn" to recognize antigens of the major histocompatibility complex (MHC; including Ia-like antigens) within the thymus (in the mouse: Zinkernagel *et al.*, 1978) and form close cell contacts with Ia$^+$ stromal cells in the human thymus (Fig. 1f; Janossy *et al.*, 1980*b*). T cells in peripheral lymphoid organs recognize foreign (*e.g.* viral) antigens in conjunction with MHC antigens according to the previous thymic experience (Zinkernagel *et al.*, 1978) and form, again, close cell contacts with strongly Ia$^+$ interdigitating reticular (IDR) cells of the T zone (Fig. 1e; Veerman, 1974; Kaiserling & Lennert, 1974; Heusermann *et al.*, 1974; Lampert *et al.*, 1980). These IDR cells are related to skin Langerhans cells and circulating "veiled" cells (Kelly *et al.*, 1978) which are also strongly Ia$^+$ (Stingl *et al.*, 1978; Spry *et al.*, 1980) and may play a role in the presentation of antigens to T cells in immunogenic form (Silberberg-Sinakin *et al.*, 1976). Another cell type which expresses Ia-like antigens is the endothelium (Hirschberg *et al.*, 1979).

Our study shows that these observations may have important pathophysiological significance. Increased expression of Ia-like antigens was found in 3 diseases: in the skin of patients with mycosis fungoides in the lymph node and skin of patients with dermatopathic lymphadenopathy (see also Lampert *et al.*, 1980) and in angio-immunoblastic lymphadenopathy (AILA). In the first 2 conditions the increased amount of Ia-like antigens was found on IDR-type cells, while in AILA the Ia-like antigens were abundant on high endothelium and arborizing vessel walls. In these conditions many T lymphoblasts formed contact with Ia$^+$ cells (Fig. 3) indicating active stimulation. Decreased expression of Ia-like antigens was seen in a thymoma taken from a patient with an autoimmune disorder. These observations show the involvement of MHC (Ia-like) antigens in a variety of diseases, and help to ask specific questions about their aetiology. In the immunostimulatory diseases the balance of T helper and suppressor cells, the function of T cells (attracting and stimulating Ia$^+$ cells?) and the function of Ia$^+$ cells (stimulating T cells?) require further analysis. Since AILA can lead to B lymphoma (Case H) or T lymphoma or "true" histiocytic lymphoma (Jones *et al.*, 1978) these studies contribute to a better understanding of pre-malignant conditions.

In conclusion, this study demonstrates that, with the availability of immunological reagents of high quality, new areas of histopathology are becoming amenable to detailed cellular analysis in tissue sections. The investigators can choose the reagent combinations which give the answer to their specific questions, and this technology seems to bridge the gap between "classical" histology and the conventional immunological (membrane-marker and functional) studies which are performed with suspensions of cells.

### REFERENCES

AISENBERG, A. C. & LONG, J. C. (1975) Lymphocyte surface characteristics in malignant lymphoma. *Am. J. Med.*, **58**, 300.

BERNARD, A., BOUMSELL, L., BAYLE, C. & 9 others (1979) Subsets of malignant lymphomas in children related to phenotype. *Blood*, **54**, 1058.

BEVERLEY, P. C. L. (1980) Production and use of monoclonal antibodies in transplantation immunology. *Proc. XI Int. Course on Transplantation and Clinical Immunology*. (In press.)

BLOOMFIELD, C. D., KERSEY, J. H., BRUNNING, R. D. & GAJL-PECZALSKA, K. J. (1976) Prognostic significance of lymphocyte surface markers in adult non-Hodgkin's malignant lymphoma. *Lancet*, ii, 1330.

BOLLUM, F. J. (1975) Antibody to terminal deoxynucleotidyl transferase. *Proc. Natl Acad. Sci., U.S.A.*, **72**, 4119.

BOLLUM, F. J. (1978) Terminal deoxynucleotidyl transferase: Biological studies. In *Advances in Enzymology*, Vol. 47. Ed. Meister. New York: John Wiley & Sons. p. 347.

BRADSTOCK, K. F., JANOSSY, G., PIZZOLO, G. & 6 others (1980) Subpopulations of normal and leukemic human thymocytes: An analysis using monoclonal antibodies. *J. Natl Canc. Inst.* (In press.)

BRAYLAN, R. C., JAFFE, E. S. & BERARD, C. W. (1975) Malignant lymphomas: Current classification and new observations. *Pathol. A.*, **10**, 213.

BRAYLAN, R. C. & RAPPAPORT, H. (1973) Tissue immunoglobulins in nodular lymphomas as compared with reactive follicular hyperplasias. *Blood*, **42**, 579.

BROOKS, D. A., BECKMAN, I., BRADLEY, J., MCNAMARA, P. J., THOMAS, M. E. & ZOLA, H. (1980) Human lymphocyte markers defined by antibodies derived from somatic cell hybrids. I. Hybridomas secreting antibody against a marker specific for human B lymphocytes. *Clin. Exp. Immunol.*, **39**, 477.

BROUET J. C., LABAUME, S. & SELIGMANN, M. (1975) Evaluation of T and B lymphocyte membrane markers in human non-Hodgkin's malignant lymphomas. *Br. J. Cancer* (Suppl. 2), **31**, 121.

FU, S. M., CHIORAZZI, N., WANG, C. Y. & 4 others (1978) Ia-bearing T lymphocytes in man. *J. Exp. Med.*, **148**, 1423.

GREAVES, M. F. & JANOSSY, G. (1978) Patterns of gene expression and the cellular origins of human leukaemias. *Biochim. Biophys. Acta*, **516**, 192.

HABESHAW, J. (1980) Investigation of non-Hodgkin lymphoma in children by surface phenotyping. *Progress in Haematology & Oncology*, Eds. Pochedly & Graham-Pole. New York: Masson Publ. Co. (In press.)

HABESHAW, J. A., CATLEY, P. F., STANSFELD, A. G. & BREARLEY, R. L. (1979). Surface phenotyping, histology and the nature of non-Hodgkin lymphoma in 157 patients. *Br. J. Cancer*, **40**, 20.

HEUSERMANN, U., STUTTE, H. J. & MULLER-HERMELINK, H. K. (1974) Interdigitating cells in white pulp of the human spleen. *Cell. Tissue Res.*, **153**, 415.

HIRSCHBERG, H., MOEN, T. & THORSBY, E. (1979) Specific destruction of human endothelial cell monolayers by anti-DRW antisera. *Transplantation*, **28**, 116.

HOFFMAN-FEZER, G., RODT, H., EULITZ, M. & THIERFELDER, S. (1976) Immunohistochemical identification of T and B lymphocytes delineated by the unlabelled antibody enzyme method. *J. Immunol. Methods*, **13**, 261.

JAFFE, E. S., SHEVACH, E. M., FRANK, M. M., BERARD, C. W. & GREEN, I. (1974) Nodular lymphoma—evidence for origin from follicular B lymphocytes. *N. Eng. J. Med.*, **290**, 813.

JANOSSY, G. (1980) Membrane markers. In *Methods in Haematology. The Leukaemia Cell*. Ed. Catovsky. Edinburgh: Churchill Livingstone. (In press.)

JANOSSY, G., BOLLUM, F. J., BRADSTOCK, K. F., MCMICHAEL, A., RAPSON, N. & GREAVES, M. F. (1979) Terminal transferase positive human bone marrow cells exhibit the antigenic phenotype of non-T, non-B acute lymphoblastic leukaemia. *J. Immunol.*, **123**, 1525.

JANOSSY, G., GOLDSTONE, A. H., CAPELLARO, D. & 4 others (1977) Differentiation linked expression of p28, 33 (Ia-like) structures on human leukaemic cells. *Br. J. Haematol.*, **37**, 391.

JANOSSY, G., HOFFBRAND, A. V., GREAVES, M. F. & 5 others (1980*a*) Terminal transferase enzyme assay and immunological membrane markers in the diagnosis of leukaemia—a multi-parameter analysis of 300 cases. *Br. J. Haematol.*, **44**, 221.

JANOSSY, G., THOMAS, J. A., BOLLUM, F. J. & 6 others (1980*b*) The human thymic microenvironment: An immunohistological study. *J. Immunol.*, **125**, 202.

JONES, D. B., CASTLEDEN, M., SMITH, J. L., MEPHAM, B. L. & WRIGHT, D. H. (1978) Immunopathology of antioimmunoblastic lymphadenopathy. *Br. J. Cancer*, **37**, 1053.

KAISERLING, E. & LENNERT, K. (1974) Die interdigitierde Retikulumzelle im menschlichen Lymphknoten: eine spezifische Zelle der thymusabhängigen Region. *Virchows Arch.* [*Cell Pathol.*], **16**, 51.

KELLY, R. H., BALFOUR, B. M., ARMSTRONG, J. A. & GRIFFITHS, S. (1978) Functional anatomy of lymph nodes. II. Peripheral lymph-borne mononuclear cells. *Anat. Rec.* **190**, 5.

KÖHLER, G. & MILSTEIN, C. (1975) Continuous cultures of fused cells secreting antibody of predefined specificity. *Nature*, **256**, 495.

KOZINER, B., FILLIPPA, D. A., MERTELSMANN, R. & 4 others (1977) Characterization of malignant lymphomas in leukemic phase by multiple differentiation markers of mononuclear cells. Correlation with clinical features and conventional morphology. *Am. J. Med.*, **63**, 556.

KUNG, P. C., GOLDSTEIN, G., REINHERZ, E. L. & SCHLOSSMAN, S. F. (1979) Monoclonal antibodies defining distinctive human T cell surface antigens. *Science*, **206**, 347.

LAMPERT, I. A., PIZZOLO, G., THOMAS, J. A. & JANOSSY, G. (1980) Immunohistochemical characterization of cells involved in dermatopathic lymphadenopathy. *J. Pathol.*, **131**, 145.

LEVY, R., WARNKE, R., DORFMAN, R. F. & HAIMOVICH, J. (1977) The monoclonality of human B-cell lymphomas. *J. Exp. Med.*, **145**, 1014.

LUKES, R. J. & COLLINS, R. D. (1974) Immunologic characterization of human malignant lymphomas. *Cancer*, **34**, 1488.

LUKES, R. J., PARKER, J. W., TAYLOR, C. R., TINDLE, B. H., CRAMER, A. D. & LINCOLN, T. L. (1978) Immunologic approach to non-Hodgkin's lymphomas and related leukaemias. Analysis of the results of multiparameter studies of 425 cases. *Semin. Hematol.*, **15**, 322.

MCMICHAEL, A. J., PILCH, J. R., GALFRE, G., MASON, D. Y., FABRE, J. W. & MILSTEIN, C. (1979) A human thymocyte antigen defined by a hybrid myeloma monoclonal antibody. *Eur. J. Immunol.*, **9**, 205.

NATHWANI, B. N., RAPPAPORT, H., MORAN, E. M., PANGALIS, G. A. & KIM, H. (1978) Malignant lymphoma arising in angioimmunoblastic lymphadenopathy. *Cancer*, **41**, 578.

PIZZOLO, G., BEVERLEY, P., SLOANE, J., BRADSTOCK, K. & JANOSSY, G. (1980) Differential diagnosis of lymphomas and anaplastic carcinoma with monoclonal antibodies in tissue sections. *Cancer*. (In press.)

REINHERZ, E. L., KUNG, P. C., GOLDSTEIN, G., LEVEY, R. H., SCHLOSSMAN, S. F. (1980) Discrete stages of human intrathymic differentiation: analysis of normal thymocytes and leukaemic blasts of T lineage. *Proc. Natl Acad. Sci U.S.A.*, **77**, 1588.

REINHERZ, E. L., KUNG, P. C., GOLDSTEIN, G. & SCHLOSSMAN, S. F. (1979) Further characterization of the human inducer T cell subset defined by monoclonal antibody. *J. Immunol.*, **123**, 2894.

RHODES, J. M., JEWELL, D. R. & JANOSSY, G. (1980) Alpha-chain disease diagnosed by rectal biopsy. *Br. Med. J.* (In press.)

SCHLOSSMAN, S. F., CHESS, L., HUMPHREYS, R. E. & STROMINGER, J. L. (1976) Distribution of Ia-like molecules on the surface of normal and leukemic human cells. *Proc. Natl Acad. Sci. U.S.A.*, **73**, 1288.

SEYMOUR, G. J., GREAVES, M. F. & JANOSSY, G. (1980) Identification of cells expressing T and p28,33 (Ia-like) antigens in sections of human lymphoid tissue. *Clin. Exp. Immunol.*, **39**, 66.

SILBERBERG-SINAKIN, I., THORBECKE, G. J., BAER, R. L., ROSENTHAL, S. A. & BEREZOWSKY, V. (1976) Antigen bearing Langerhans cells in skin, dermal lymphatics and in lymph nodes. *Cell. Immunol.*, **25**, 137.

SPRY, C. J. F., PFLUG, J., JANOSSY, G. & HUMPHREY, J. H. (1980) "Veiled" cells with "Ia-like" membrane antigens in human afferent lymph. *Clin. Exp. Immunol.*, **39**, 750.

STEIN, H., PETERSEN, N., GAEDICKE, G., LENNERT, K. & LANDBECK, G. (1976) Lymphoblastic lymphoma of convoluted or acid phosphatase type: A tumour of T precursor cells. *Int. J. Cancer*, **17**, 292.

STEIN, H., SIEMSSEN, U. & LENNERT, K. (1978) Complement receptor subtypes C3b and C3d in lymphatic tissue and follicular lymphoma. *Br. J. Cancer*, **37**, 520.

STINGL, G., KATZ, S. I., CLEMENT, L., GREEN, I. & SHEVACH, E. M. (1978) Immunologic functions of Ia-bearing epidermal Langerhans cells. *J. Immunol.*, **121**, 2005.

TAYLOR, C. R. (1978) Immunoperoxidase techniques: Theoretical and practical aspects. *Arch. Pathol.*, **102**, 113.

THIERFELDER, S., RODT, H. & THIEL, E. (Eds) (1977) *Immunological Diagnosis of Leukemias and Lymphomas*. Berlin: Springer-Verlag.

VEERMAN, A. J. P. (1974) On the interdigitating cells in the thymus-dependent area of the rat spleen: A relation between the mononuclear phagocyte system and T lymphocytes. *Cell Tissue Res.*, **148**, 247.

WALDRON, J. A., LEECH, J. H., GLICK, A., FLEXNER, J. M. & COLLINS, R. D. (1977) Malignant lymphoma of peripheral T-lymphocyte origin: Immunologic, pathologic and clinical features in six patients. *Cancer*, **40**, 1604.

WARNKE, R. & LEVY, R. (1978) Immunopathology of follicular lymphomas: A model of B-lymphocytic homing. *N. Engl. J. Med.*, **298**, 481.

WINCHESTER, R. J., ROSS, G. D., JAROWSKI, C. I., WANG, C. Y., HALPER, J. & BROXMEYER, H. E. (1977) Expression of Ia-like antigen molecules on human granulocytes during early phases of differentiation. *Proc. Natl Acad. Sci. U.S.A.*, **74**, 4012.

ZINKERNAGEL, R. M., CALLAHAN, G. N., ALTHAGE, A., COOPER, S., KLEIN, P. A. & KLEIN, J. (1978) On the thymus in the differentiation of "H-2 self-recognition" by T cells: Evidence for dual recognition? *J. Exp. Med.*, **147**, 882.